# The ESCRT and autophagy machineries cooperate to repair ESX-1-dependent damage at the *Mycobacterium*-containing vacuole but have opposite impact on containing the infection

**DOI:** 10.1371/journal.ppat.1007501

**Published:** 2018-12-31

**Authors:** Ana T. López-Jiménez, Elena Cardenal-Muñoz, Florence Leuba, Lilli Gerstenmaier, Caroline Barisch, Monica Hagedorn, Jason S. King, Thierry Soldati

**Affiliations:** 1 Department of Biochemistry, Faculty of Science, University of Geneva, Sciences II, Geneva, Switzerland; 2 Section Parasitology, Bernhard Nocht Institute for Tropical Medicine, Hamburg, Germany; 3 Life Sciences and Chemistry, Jacobs University Bremen gGmbH, group Ribogenetics, Bremen, Germany; 4 Department of Biomedical Science, University of Sheffield, Western Bank, Sheffield, United Kingdom; University of Washington, UNITED STATES

## Abstract

Phagocytic cells capture and kill most invader microbes within the bactericidal phagosome, but some pathogens subvert killing by damaging the compartment and escaping to the cytosol. To prevent the leakage of pathogen virulence and host defence factors, as well as bacteria escape, host cells have to contain and repair the membrane damage, or finally eliminate the cytosolic bacteria. All eukaryotic cells engage various repair mechanisms to ensure plasma membrane integrity and proper compartmentalization of organelles, including the Endosomal Sorting Complex Required for Transport (ESCRT) and autophagy machineries. We show that during infection of *Dictyostelium discoideum* with *Mycobacterium marinum*, the ESCRT-I component Tsg101, the ESCRT-III protein Snf7/Chmp4/Vps32 and the AAA-ATPase Vps4 are recruited to sites of damage at the *Mycobacterium*-containing vacuole. Interestingly, damage separately recruits the ESCRT and the autophagy machineries. In addition, the recruitment of Vps32 and Vps4 to repair sterile membrane damage depends on Tsg101 but appears independent of Ca^2+^. Finally, in absence of Tsg101, *M*. *marinum* accesses prematurely the cytosol, where the autophagy machinery restricts its growth. We propose that ESCRT has an evolutionary conserved function to repair small membrane damage and to contain intracellular pathogens in intact compartments.

## Author summary

Upon uptake by a host cell, intracellular pathogens reside in a membranous compartment called phagosome. Within the phagosome, microbes are protected from the extracellular and cytosolic immune defences, whilst access to nutrients is limited. Some microbes gain access to the host cytosol by damaging the membrane of the phagosome, a step preceding egress and dissemination. Autophagy, a major catabolic pathway in eukaryotes, has been previously proposed to contribute to autonomous cell defence and to repair the membrane damage induced by intracellular pathogens. Here, we provide evidence that, in *Dictyostelium discoideum*, autophagy does not work alone in the containment of vacuolar mycobacteria, but it operates together with the Endosomal Sorting Complex Required for Transport (ESCRT), a protein machinery recently shown to repair endolysosomal damage. We demonstrate that the membrane perforations induced by the ESX-1 secretion system of *Mycobacterium marinum* are targeted by both ESCRT and autophagy, which seal the damaged vacuole. We propose that ESCRT might mend small membrane pores, whilst autophagy patches larger cumulative wounds. Interestingly, and contrary to what has been described in mammalian cells for ESCRT-dependent endolysosomal repair, in *D*. *discoideum*, repair of sterile membrane damage appears not to require Ca^2+^. The evolutionary conservation of the function of ESCRT in membrane repair suggests that this machinery plays an ancestral and widespread role to contain a broad range of intracellular pathogens.

## Introduction

After phagocytic uptake, the closely related pathogenic bacteria *Mycobacterium tuberculosis* and *M*. *marinum* reside in an altered and maturation-arrested phagosome, thereby avoiding its toxic chemical environment [1], but remaining protected from the cell-autonomous cytosolic defences [2]. This *Mycobacterium*-containing vacuole (MCV) becomes permissive for the bacilli to survive and replicate [3, 4]. However, bacteria access to nutrients is limited. To circumvent this restriction, tubercular mycobacteria damage the MCV and escape to the cytosol. The site of MCV rupture becomes a complex battlefield where various machineries cooperate to repair membrane damage and control cytosolic bacteria. Here, we used the *Dictyostelium discoideum-M*. *marinum* system to study the role of Endosomal Sorting Complex Required for Transport (ESCRT) and autophagy in membrane repair during both sterile and pathogen-induced damage. We show that the function of ESCRT-III in membrane repair is evolutionarily conserved, that it contributes to the integrity of the MCV and plays an unrecognised role in cell-autonomous defence. We also provide evidence that the ESCRT-III and autophagy pathways act in parallel to repair endomembrane compartments, but differ in their ability to restrict mycobacteria growth in the cytosol of infected cells.

To access the cytosol, mycobacteria make use of a crucial pathogenicity locus, the Region of Difference 1 (RD1), which encodes the ESX-1 system responsible for the secretion of the membranolytic peptide ESAT-6 [5]. Together with the mycobacterial branched apolar lipids phthiocerol dimycocerosates (PDIMs) [6, 7], ESAT-6 produces membrane perforations that cause MCV rupture and bacterial escape to the cytosol [3, 8, 9], a step that precedes egress of the bacteria and their dissemination to neighboring cells (reviewed in [10, 11]). At the site of MCV rupture cells need to discriminate self from non-self as well as from topologically misplaced self molecules. Damage exposes either pathogen-associated molecular patterns (PAMPs) or damage-associated molecular patterns (DAMPs, from the vacuole) to cytosolic machineries that sense them, resulting in the deposition of “repair-me” and "eat-me" signals. Among the latter, the best studied during infection by various intracellular bacteria, including mycobacteria, is ubiquitin. It is conjugated to mycobacterial or host proteins by the E3 ligases NEDD4 [12], Smurf1 [13], Parkin [14] and TRIM16 [15] leading to the recruitment of the autophagy machinery to restrict their growth (xenophagy). In addition, mammalian galectins such as Gal-3 [15, 16], Gal-8 [17] and Gal-9 [18, 19] bind to exposed lumenal glycosylations of damaged endosomes [20, 21] or to components of the cell wall of bacterial pathogens, and thus might play a defence role against mycobacterial infection. Although the signaling that leads from these “eat-me” tags to the recruitment of autophagy is well understood [2, 22], how the recruitment of the repair machinery(ies) is mediated remains mysterious [23].

Strikingly, autophagy has been shown to participate in restricting the growth of some bacteria [23, 24], whilst also contributing to the establishment and maintenance of the compartments containing *Salmonella* Typhimurium [25] and *M*. *marinum* [23]. Recent studies have highlighted the potential of another cytosolic machinery, the ESCRT, in both repair of membranes damaged by sterile insults [26–29] and in the control of mycobacteria infection [30]. ESCRT is an evolutionary-conserved machinery, composed of four protein complexes (ESCRT-0, -I, -II, -III), the AAA-ATPase Vps4 and multiple accessory proteins, involved in various processes of membrane remodeling. This is achieved by the ESCRT-III component Chmp4/Snf7/Vps32, which polymerizes in spirals that drive membrane deformation [31], whereas Vps4 triggers membrane scission and ESCRT-III disassembly [32–34]. The most studied functions are in the invagination of intralumenal vesicles of multivesicular bodies, which is initiated by the ubiquitination of the cargo to be sorted [35], in the release of viruses by budding off the plasma membrane [36] and in the constriction of the cytokinetic bridge during mitosis [37]. Additional roles for ESCRT have been uncovered, such as exosome and microvesicle biogenesis, neuron pruning, removal of defective nuclear pores, and micro- and macroautophagy [38]. Importantly, ESCRT-III has also recently been proposed to mediate repair at a number of membranes such as plasma membrane wounds of less than 100 nm, possibly by surface extrusion [26], as well as small disruptions in endolysosomes [28, 29]. The ESCRT complexes are implicated in infection of *Drosophila* S2 cells with *M*. *fortuitum* [39] and *Listeria monocytogenes* [40]. Follow-up studies showed that depletion of the ESCRT proteins Vps28 or Tsg101 led to an increase of *M*. *smegmatis* proliferation in RAW macrophages. These bacteria were found to be highly ubiquitinated at 3 hours post-infection (hpi) in S2 cells, and electron microscopy (EM) inspection revealed that most of them were inside a vacuolar compartment. In the case of *L*. *monocytogenes*, a mutant unable to secrete the pore-forming toxin LLO, but still expressing two membranolytic phospholipases C, was found to escape more to the cytosol in S2 cells devoid of several ESCRT proteins (Bro/ALIX/AlxA, Vps4 and Snf7/Chmp4/Vps32) [41]. In this context, ESCRT was involved in membrane trafficking and the establishment of the pathogen-containing compartment. However, whether ESCRT plays a role in the repair of damage inflicted by the mycobacteria remains to be addressed.

Recent reports brought evidence for a role of ESCRT-III in repairing sterile damages to various membranes, such as the plasma membrane (laser wounding, detergents, pore-forming toxins) [26, 27], the nuclear membrane (laser wounding and confined cell migration) [42, 43] and endolysosomes (lysosomotropic compounds and silica crystals) [28, 29]. Importantly, a recent report also showed the recruitment of the ESCRT-III component Chmp4B to vacuoles containing *Coxiella burnetti* [29]. ESCRT-III recruitment to damage at the plasma membrane and lysosomes was proposed to depend on the recognition of a local increase of Ca^2+^ by ALIX and/or ALG2 [26–28]. In all other cases, ESCRT-III is recruited to the disrupted membranes, but the cues and signaling pathways involved remain unclear.

The ESCRT and autophagy machineries are highly conserved in the social amoeba *D*. *discoideum* [44]. In the last decade, *D*. *discoideum* has emerged as a powerful model system to study host-pathogen interactions, including with the human pathogens *Legionella pneumophila*, *Pseudomonas aeruginosa*, *Vibrio cholerae*, as well as various mycobacteria species such as *M*. *tuberculosis* and *M*. *marinum* (reviewed in [45]).

## Results

### The ultrastructure of the MCV at the site of rupture reveals a complex battlefield between *M*. *marinum* and its host

**Fig 1:**
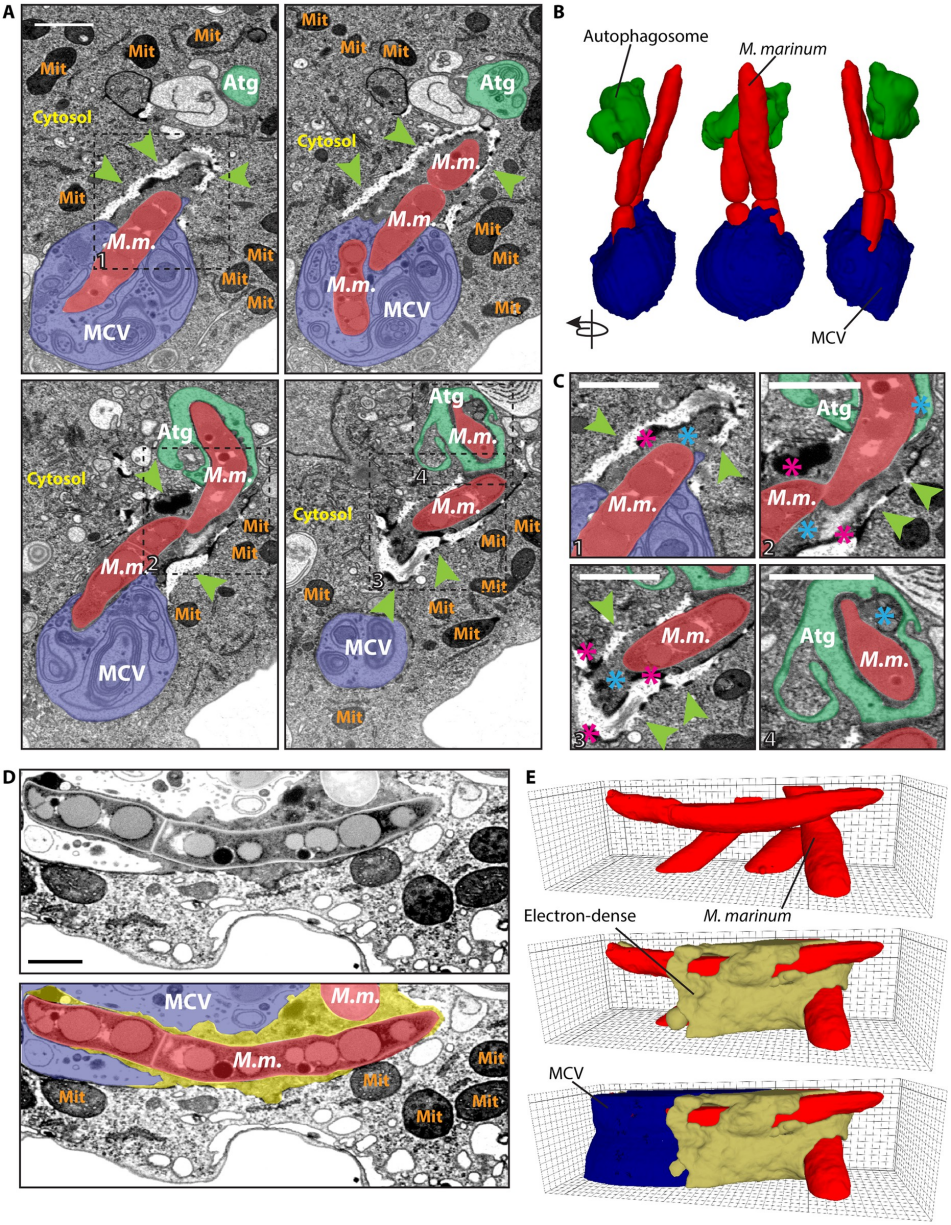
The ultrastructure of the MCV at the point of rupture reveals a complex battlefield between the *M*. *marinum* and its host. *D*. *discoideum* was infected with *M*. *marinum* and fixed at 24 hpi for visualization by FIB-SEM. (A) Sections of the same infected cell, showing a disrupted MCV (blue), *M*. *marinum* (red) accessing the cytosol and a potential autophagosome (green) engulfing the pole of a cytosolic bacterium. Green arrowheads point to a complex structure with discontinuities and electron-dense boundaries surrounding the cytosolic *M*. *marinum*. Squares delimitate the areas of interest magnified in C. (B) 3D reconstruction of the FIB-SEM stack shown in A. (C) The cytosolic bacteria were surrounded by a structure (green arrowheads) with very electron-dense boundaries (pink asterisks). The cytosolic material between the bacteria and this structure or the autophagosome was slightly more electron-dense than the rest of the cytosol (blue asterisks) (D). Section of a cell, showing a disrupted MCV (blue), *M*. *marinum* (red) and the dark electron-dense material surrounding the sites of escape (blue asterisks, yellow). (E) 3D reconstruction of the FIB-SEM stack shown in D (see also S1 Movie). Abbreviations: *M*. *m*., *M*. *marinum*; MCV, *Mycobacterium*-containing vacuole; Mit, Mitochondria; Atg, Autophagosome. Scale bars, 1 μm.

Previous work demonstrated that the MCV suffers continuous ESX-1-dependent insults and injuries during infection, from the first hour post-entry, when no macroscopic sign of breakage is observed, until about 24 hpi, when the bacteria escape to the cytosol [3]. Ubiquitination is among the first readout of damage and was shown to trigger the recruitment of the classical autophagy machinery components such as Atg18, p62 and Atg8 [23]. In order to gain a deeper morphological and ultrastructural insight into the sites of MCV membrane damage during *M*. *marinum* infection, cells at 24 hpi were subjected to Focus Ion Beam Scanning Electron Microscopy (FIB-SEM) (Fig 1). This revealed a complex interface between the MCV and the cytosol at the site of bacteria escape. Fig 1A, 1B and 1C show views and 3D reconstructions of bacteria escaping from an MCV and captured by an autophagosome. The zone surrounding the portion of the bacteria in contact with the cytosol shows both discontinuities and highly electron-dense material (Fig 1C and 1D). Careful inspection of the samples, together with their 3D reconstruction, revealed that this compact mass was apparently not separated from the MCV content or the host cytosol by any membrane (Fig 1D and 1E, S1 Movie). These observations suggest that bacterial and host factors accumulate at the place of MCV rupture. In macrophages and *D*. *discoideum*, damaged MCVs and escaping *M*. *marinum* accumulate ubiquitin, although the substrate of ubiquitination remains elusive [23, 46, 47]. Therefore, we speculate that the dark electron-dense material observed at the areas of MCV rupture might correspond to local accumulation of ubiquitinated cargoes, proteins belonging to the autophagy pathway and possibly other cytosolic machineries, such as the ESCRT, recently implicated in endolysosomal membrane damage repair [28].

### ESCRT is recruited to the MCV upon *M*. *marinum*-induced damage

**Fig 2:**
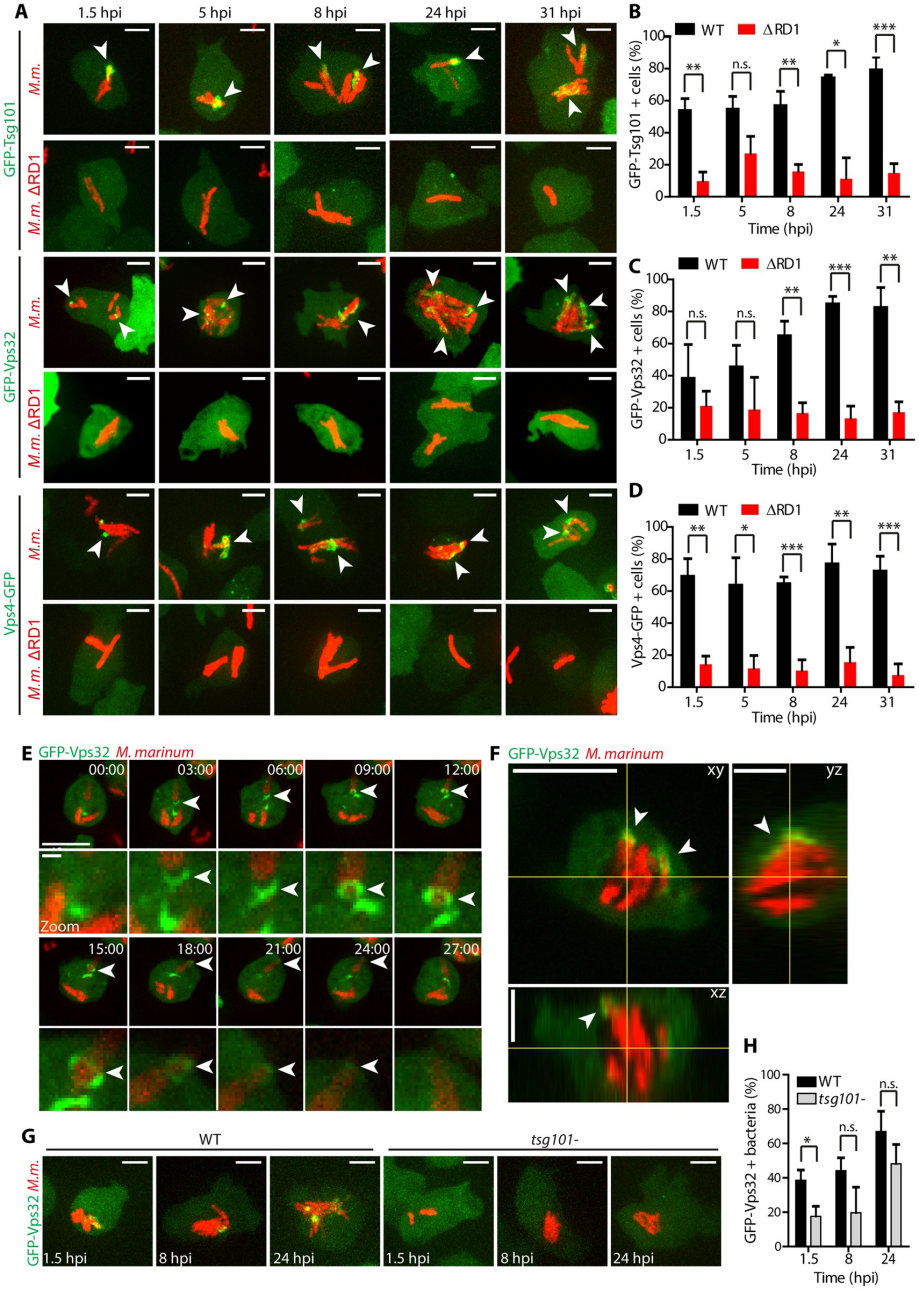
Recruitment of ESCRT components to the MCV upon *M*. *marinum* induced damage. (A) *D*. *discoideum* expressing GFP-Tsg101, GFP-Vps32 or Vps4-GFP were infected with *M*. *marinum* wt or *M*. *marinum* ΔRD1, and z-stacks were acquired at 1.5, 5, 8, 24 and 31 hpi. Maximum projections are shown. GFP-Tsg101, GFP-Vps32 and Vps4-GFP structures (white arrows) appeared in the vicinity of the MCV containing *M*. *marinum* wt (red), and to a lesser extent around *M*. *marinum* ΔRD1 (red). Scale bars, 5 μm. (B-D) Quantification of GFP-Tsg101 structures (patches and foci) and GFP-Vps32 and Vps4-GFP structures (patches and rings) in the vicinity of *M*. *marinum* wt or *M*. *marinum* ΔRD1. Plots show the mean and standard deviation (GFP-Tsg101 1.5, 8, 24, 31 hpi N = 3, 38≤n≤198; 5 hpi N = 2, 59≤n≤117, GFP-Vps32 N = 3 32≤n≤145; Vps4-GFP N = 3, 64≤n≤233). (E) *D*. *discoideum* expressing GFP-Vps32 were infected with *M*. *marinum* wt (red) and monitored by time-lapse microscopy every 3 min (see also S2 Movie). Maximum projections of the same cell are shown (time indicated in the top right corner). GFP-Vps32 rings formed and appeared to move along the bacterium (arrows). Bottom panels show insets focused on the ring structures (arrows). Scale bars, 10 μm and 1 μm for the insets. (F) Section of a z-stack showing the recruitment of GFP-Vps32 to the vicinity of the MCV (bacteria in red) at 31 hpi. Projections of the xz and yz planes are shown. Scale bar, 10 μm. (G) *D*. *discoideum* wt or *tsg101-* expressing GFP-Vps32 were infected with *M*. *marinum* (in red) and z-stacks were acquired at 1.5, 8 and 24 hpi. Maximum projections are shown. GFP-Vps32 was recruited to a lesser extent to the MCV in *tsg101-* cells. Scale bars, 5 μm. (H) Quantification of GFP-Vps32 structures formed in the vicinity of *M*. *marinum* in wt or *tsg101-* cells. The plot shows the mean and standard deviation (N = 3, 76≤n≤154). Two-way ANOVA and post hoc Fisher’s LSD tests were performed.

One of the first host responses to membrane damage is the ubiquitination of the bacilli and the broken MCV, followed by recruitment of the autophagy machinery to delay escape to the cytosol [23]. To test whether the ESCRT machinery is also recruited to damaged MCVs, cells expressing the ESCRT-I component GFP-Tsg101, the ESCRT-III effector GFP-Vps32 or the ATPase Vps4-GFP were infected with wild-type (wt) *M*. *marinum* or *M*. *marinum* ΔRD1 (Fig 2A–2D). All three proteins were recruited to MCVs containing wt *M*. *marinum*, but were significantly less so in cells infected with the attenuated *M*. *marinum* ΔRD1. This bacterial strain causes very limited membrane damage and escapes very inefficiently to the cytosol, due to the lack of secretion of ESAT-6 but despite the presence of similar level of PDIMs compared to wt bacteria [6, 48] (S1 Fig). The ESCRT-positive structures comprised small foci, patches of several micrometers and even rings that were observed to slide along the length of the bacteria compartment (Fig 2E and S2 Movie). These structures seemed to become larger at later time-points (Fig 2A, 24 and 31 hpi), suggesting increased recruitment upon cumulative damage as infection progresses. Careful 3D inspection at late time-points revealed that GFP-Vps32 patches always surrounded the MCV, but were not in its lumen (Fig 2F). During membrane remodeling in mammalian cells, the ESCRT-III complex can be recruited to biological membranes via several pathways, one of the most studied relies on the ESCRT-I component Tsg101 [49]. Importantly, in cells lacking Tsg101, GFP-Vps32 structures were significantly reduced at early times of infection (Fig 2G and 2H), which may indicate that *M*. *marinum*-induced damage triggers one or more pathways of ESCRT-III recruitment to the MCV. Altogether, we conclude that the ESCRT-III is recruited to the MCV in an ESX-1 dependent manner, consistent with a role in membrane repair.

### ESCRT-III and autophagy are recruited to the disrupted MCV at spatially distinct sites

**Fig 3:**
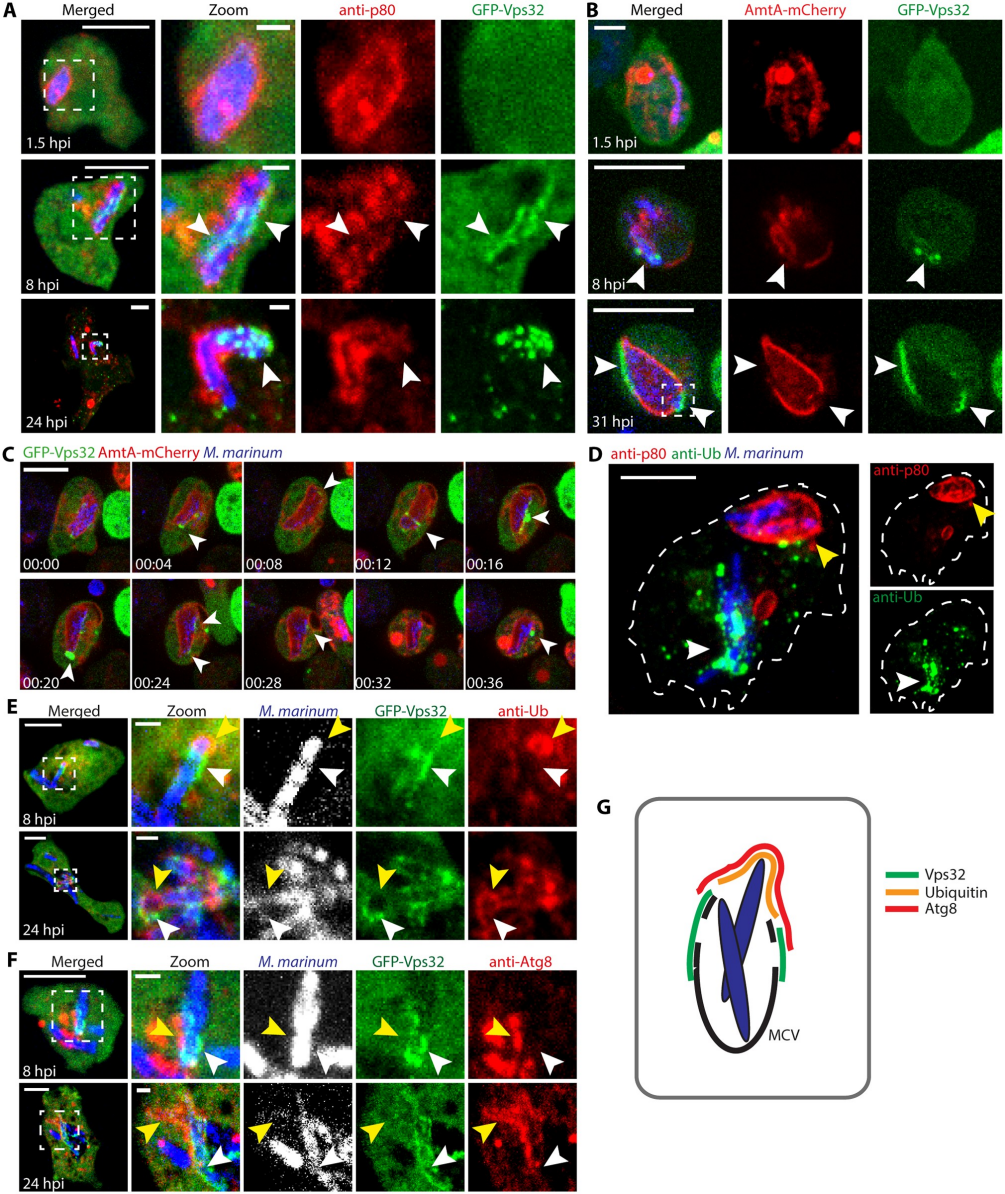
GFP-Vps32 and GFP-Atg8 are recruited to the disrupted MCV at spatially distinct sites. (A) *D*. *discoideum* expressing GFP-Vps32 were infected with *M*. *marinum* (blue) and fixed for immunostaining against p80 to label the MCV (red). At 1.5 hpi, no GFP-Vps32 structure was visible at apparent intact MCVs. At 8 and 24 hpi, GFP-Vps32 foci and patches appeared in the vicinity of the bacteria, where the MCV was evidently disrupted (arrows). Scale bar, 10 μm and 1 μm for the insets. (B-C) *D*. *discoideum* expressing GFP-Vps32 and AmtA-mCherry were infected with *M*. *marinum* (blue) and monitored by time-lapse microscopy. (B) Still images show GFP-Vps32 patches on the disrupted MCV (arrows) (see also S2A–S2C Fig). (C) Time-lapse showing the dynamics of association of GFP-Vps32 to the AmtA-mCherry labelled MCV (see also S3 Movie). Scale bars, 10 μm and 1 μm for the insets. (D) *D*. *discoideum* was infected with *M*. *marinum* and fixed for immunostaining at 24 hpi. Only bacteria (blue) that have escaped the MCV (p80, red) showed ubiquitin structures (green). Scale bar, 5 μm. (E-F) *D*. *discoideum* expressing GFP-Vps32 were infected with *M*. *marinum* (blue) and fixed for immunostaining at 8 and 24 hpi to visualize ubiquitin or Atg8 (red). GFP-Vps32 and ubiquitin or Atg8 were recruited to the same macroscopic region of the MCV, but they did not perfectly colocalise. GFP-Vps32 formed patches devoid of ubiquitin or Atg8 staining (white arrows). Vice versa, ubiquitin and Atg8 appeared in areas where no GFP-Vps32 was observed (yellow arrows). Scale bar, 5 μm and 1μm for the insets. (G) Schematic representation of the Vps32, ubiquitin and Atg8 recruitments at the damaged MCV.

In order to gain a deeper insight on the GFP-Vps32 structures observed during *M*. *marinum* infection, the precise localization of GFP-Vps32 on the MCV was analyzed. The MCV membrane was visualised by the presence of the ammonium transporter AmtA-mCherry, or antibodies against the predicted copper transporter p80 [23] (Fig 3A and 3B). MCVs with an apparent continuous staining for p80 or AmtA-mCherry (Fig 3A and 3B, 1.5 hpi) were not associated with GFP-Vps32. On the contrary, compartments that displayed clear disrupted staining for p80 or AmtA-mCherry presented numerous GFP-Vps32 patches at the sites of membrane wounds (Fig 3A and 3B, 8, 24 and 31 hpi). Close inspection of these images revealed that, at sites of GFP-Vps32 recruitment, the damaged MCV membrane was sometimes invaginated towards the lumen of the compartment, away from the cytosol (S2A Fig). These invaginations are reminiscent of the membrane deformations observed sometimes at the MCV by EM, which are surrounded by dense cytosolic material (S2B and S2C Fig). Time-lapse microscopy enabled tracking of the GFP-Vps32 structures associated with the MCV, indicating direct assembly onto the membrane of the compartment rather than delivery via pre-existing structures (Fig 3C). GFP-Vps32 structures remained associated with the MCV for several minutes (Fig 3C and S3 Movie).

*M*. *marinum* in intact MCVs stained by p80 are rarely ubiquitinated, contrary to bacteria in the cytosol (Fig 3D). Therefore, we wondered whether Vps32 would be recruited at sites of ubiquitination, together with the main autophagy marker Atg8. Remarkably, all three proteins localized at disrupted MCVs, but the level of colocalisation of Vps32 with ubiquitin (Fig 3E) or Atg8 (Fig 3F) was limited. Instead, GFP-Vps32 seemed to be recruited more proximally to the membrane remnants of the MCV than ubiquitin and Atg8, which predominantly decorated the bacteria poles fully exposed to the cytosol. Besides, at the boundary between the zones enriched in GFP-Vps32 and Atg8, GFP-Vps32 was more proximal to the bacteria, possibly indicating an earlier recruitment (Fig 3E–3G). Taken all together, recruitment of ESCRT-III proteins to the *M*. *marinum* MCV seems to happen earlier and at different places than the autophagic recognition of the bacteria, suggesting that ESCRT-III and the autophagy pathway might play separate functions in repair and additionally in xenophagic capture.

### Differential spatial and temporal recruitment of ESCRT and autophagy upon sterile damage

**Fig 4:**
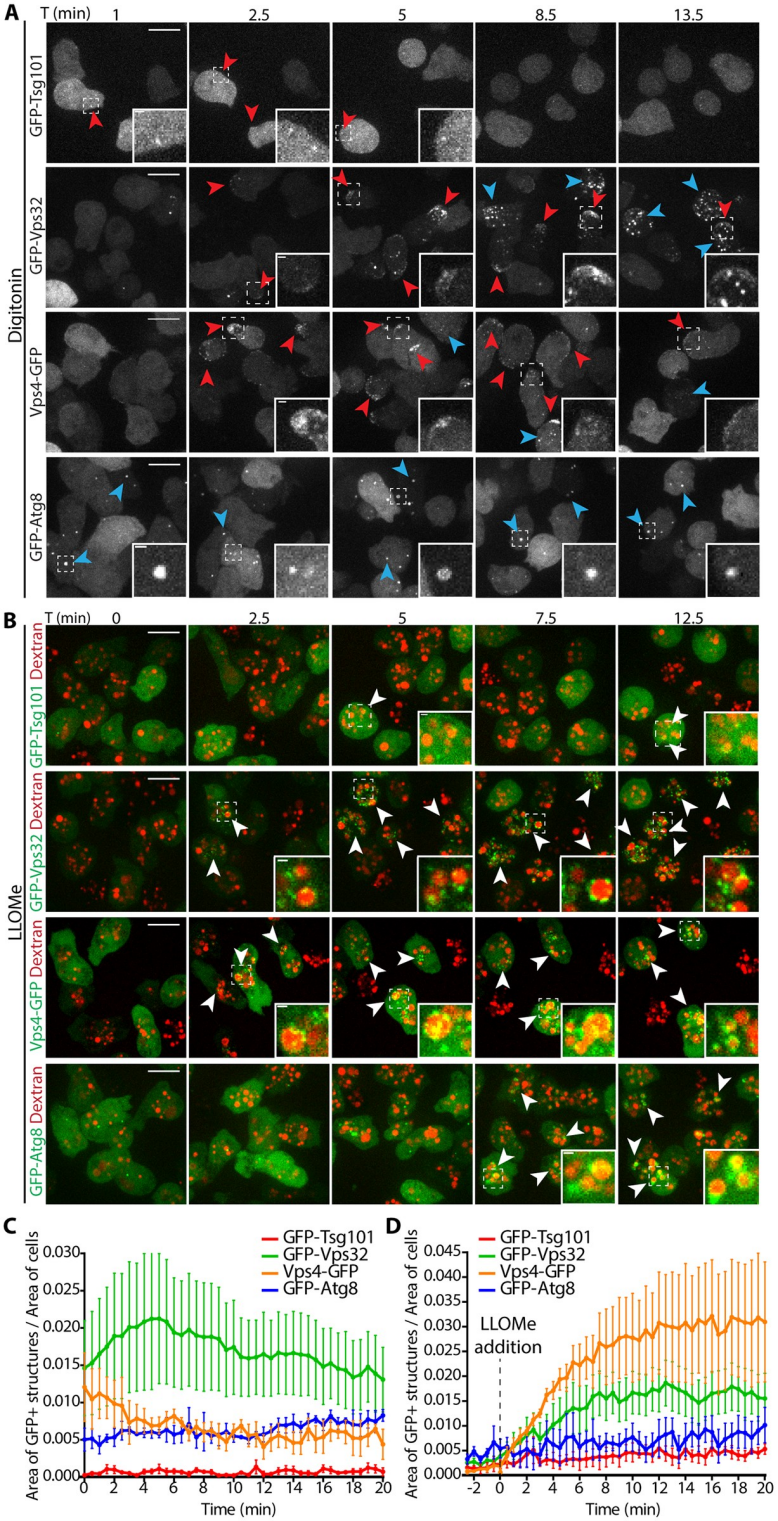
Differential spatial and temporal recruitment of ESCRT components and Atg8 upon sterile damage. *D*. *discoideum* expressing GFP-Tsg101, GFP-Vps32, Vps4-GFP or GFP-Atg8 were subjected to membrane disrupting agents and monitored by time-lapse imaging. (A) Treatment with digitonin led to the appearance of the three ESCRT-components but not GFP-Atg8 at the plasma membrane within minutes (see also S4 Movie). (B) Cells were incubated with 10 kDa fluorescent dextran for at least 3 h to label all endosomal compartments and treated with LLOMe (see also S5 Movie). Punctate ESCRT-structures appeared at the periphery of the lysosomes as early as 2.5 min after the addition of LLOMe. More diffuse, ring-like GFP-Atg8 structures were visible about 5 min later. Scale bars, 10 μm or 1 μm for the insets. (C-D) Quantification of the time-lapse experiments shown in (A-B), respectively. The proportion of the cell area occupied by the structures in maximum projections is plotted as a function of time. Graphs show the mean and SEM of N≥3 independent experiments.

Mammalian ESCRT and autophagy machineries localize to damaged membranes for the repair of wounds and removal of terminally incapacitated organelles, respectively [26–29, 42, 43]. To test whether components of both machineries were also involved in membrane repair in *D*. *discoideum*, cells expressing GFP-Tsg101, GFP-Vps32 or Vps4-GFP, as well as GFP-Atg8 were subjected to membrane damaging agents, such as the detergent digitonin or the lysosome-disrupting agent Leu-Leu-O-Me (LLOMe) (Fig 4). Digitonin inserts first into the sterol-rich plasma membrane and then, upon endocytosis, reaches the endosomes. Consistent with this, digitonin initially induced at the plasma membrane dots and crescent-shaped structures of GFP-Vps32 and Vps4-GFP (Fig 4A and 4C, S4 Movie). After a few minutes, dispersed foci appeared throughout the cytoplasm, suggesting progressive disruption of endomembranes. These structures were very dynamic and continued forming for many minutes after onset of the treatment (Fig 4C). In agreement with a role of ESCRT-III in repair, discreet foci of the ESCRT-I component Tsg101 were also observed in the vicinity of the plasma membrane with a similar timing but much reduced size and frequency (Fig 4C), supporting a role upstream of the recruitment of the ESCRT-III effectors. In contrast, treatment with digitonin did not lead to the recruitment of GFP-Atg8 to the plasma membrane, but it remained at a roughly constant level in the autophagosomal compartment over time (Fig 4A and 4C, S4 Movie). These results support a role for ESCRT but not autophagy in plasma membrane repair of this type of wound. On the other hand, LLOMe induced the formation of both ESCRT- (GFP-Tsg101, GFP-Vps32 and Vps4-GFP) and autophagy- (GFP-Atg8) positive structures at the periphery of lysosomes labelled with fluorescent dextran (Fig 4B and 4D, S5 Movie). The structures were diverse in morphology and dynamics. GFP-Tsg101, GFP-Vps32 and Vps4-GFP appeared almost immediately as discrete foci surrounding lysosomes, and again, GFP-Tsg101 structures were significantly reduced in size compared to GFP-Vps32 and Vps4-GFP, suggesting it functions in the initiation of the pathway. In contrast, GFP-Atg8 formed a more continuous ring that became apparent only several minutes later (Fig 4D). To further analyse the temporal dynamics of both ESCRT-III and autophagy machineries on disrupted lysosomes, *D*. *discoideum* co-expressing RFP-Vps32 and GFP-Atg8 were treated with LLOMe (S3A Fig and S6 Movie). RFP-Vps32 foci forming circular structures were visible before the appearance of GFP-Atg8. This spatial appearance and partial temporal segregation suggest an independent involvement of ESCRT-III and autophagy during lysosome damage. To confirm and extend the involvement of ESCRT-III in membrane repair in *D*. *discoideum*, cells expressing GFP-Vps32 or Vps4-GFP were monitored while exposed to other sterile damage. The lysosomotrophic agent glycyl-L-phenylalanine 2-naphthylamide (GPN) induced similar structures as LLOMe (S3B Fig). We noticed that, the structures formed by GFP-Vps32, known to build the polymers that deform membranes [31] were large and intense (Fig 4 and S3B Fig), and especially long-lived on injured lysosomes, where several foci remained in close apposition to dextran-labelled compartments for several minutes (Fig 4 and S3C and S3D Fig). Sometimes Vps4-GFP structures were less obvious but quantifications confirmed their clear presence, and they were also long-lived (Fig 4 and S3B Fig). In contrast, GFP-Tsg101 structures were less frequent, less intense and short-lived (Fig 4), consistent with its upstream role in recruiting the membrane-remodelling ESCRT-III.

### Mechanistic characterization of ESCRT-III recruitment at the sites of sterile damage

**Fig 5:**
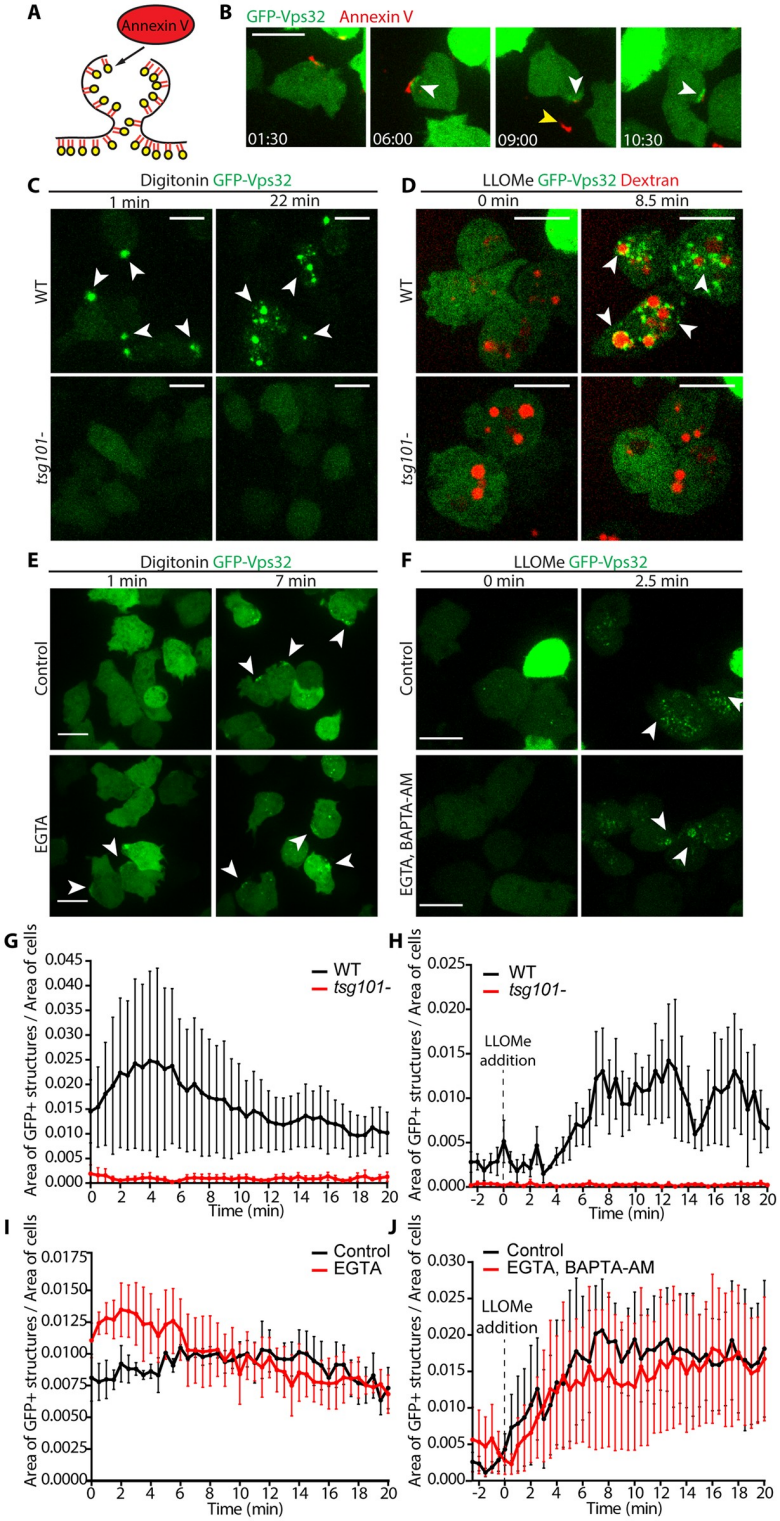
Mechanistic characterization of ESCRT-III and autophagy recruitment and repair of membrane damage. (A) Schematic representation of the experiment shown in B. Annexin V binds to PS exposed upon plasma membrane disruption. (B) *D*. *discoideum* were incubated with Annexin V Alexa Fluor 594 conjugate in the presence of Ca^2+^ and then treated with digitonin and monitored by time-lapse imaging (see also S5 Movie). GFP-Vps32 structures (in green) appeared in close proximity of Annexin V-positive structures (in red, white arrows). At later times, the Annexin V-positive structure was released into the medium (yellow arrows), leaving a GFP-Vps32 “scar” (white arrow). Time is indicated in the bottom left corner. (C-D) In cells lacking Tsg101, neither digitonin nor LLOMe treatment led to the formation of GFP-Vps32 structures. (D) Endosomes (in red) were labelled with 10 kDa fluorescent dextran for at least 3 h. (E) Cells were incubated with EGTA or mock-incubated, treated with digitonin and monitored by time-lapse microscopy. Neither spatial nor temporal differences in GFP-Vps32 recruitment (white arrows) were observed. (F) Cells were incubated with EGTA and BAPTA-AM or mock-incubated, treated with LLOMe and monitored by time-lapse microscopy. Neither spatial nor temporal differences in GFP-Vps32 recruitment (white arrows) were observed. Scale bars correspond to 10 μm. (G-J) Quantification of the time-lapse experiments shown in (C-F), respectively. The proportion of the cell area occupied by the structures in maximum projections is plotted as a function of time. Graphs show the mean and SEM of N = 3 independent experiments.

To confirm that the ESCRT-III structures formed at the site of membrane repair, cells expressing GFP-Vps32 were treated with digitonin in the presence of fluorescently-labelled Annexin V to reveal exofacially exposed phosphatidyl-serine (PS) (Fig 5A and 5B and S7 Movie). The majority of the GFP-Vps32 crescent structures were also labelled with Annexin V (Fig 5B). The Annexin V-positive structures were released to the extracellular medium, suggesting that damaged membranes were extruded instead of internalized.

In mammalian cells, ESCRT-III can be recruited to membranes by at least three mechanisms depending on the identity of the membrane and the specific role exerted by ESCRT (reviewed in [49]). In *D*. *discoideum* cells lacking Tsg101, GFP-Vps32 structures were almost completely abolished upon digitonin or LLOMe treatments (Fig 5C, 5D, 5G and 5H), providing a strong evidence that Tsg101 lies upstream of ESCRT-III during membrane repair caused by these types of sterile damage.

It has been proposed that the local increase of intracellular Ca^2+^ upon membrane damage recruits ESCRT-III to the plasma and lysosomal membranes in HeLa cells and myoblasts [26–28]. To test whether the formation of GFP-Vps32 structures also relied on a Ca^2+^-mediated signaling in *D*. *discoideum*, cells were treated with digitonin in the presence of the non-permeant Ca^2+^ chelator EGTA, or with LLOMe in the presence of EGTA and the cell-permeant BAPTA-AM (Fig 5E, 5F, 5I and 5J). In both cases, GFP-Vps32 structures appeared at the wound site with very similar morphology, size and dynamics. In conclusion, in *D*. *discoideum*, ESCRT-III recruitment to membranes damaged by these sterile agents was Tsg101-dependent but appears independent of Ca^2+^ signalling.

### Cells lacking Tsg101 or Atg1 are defective at maintaining lysosome integrity upon LLOMe treatment

**Fig 6:**
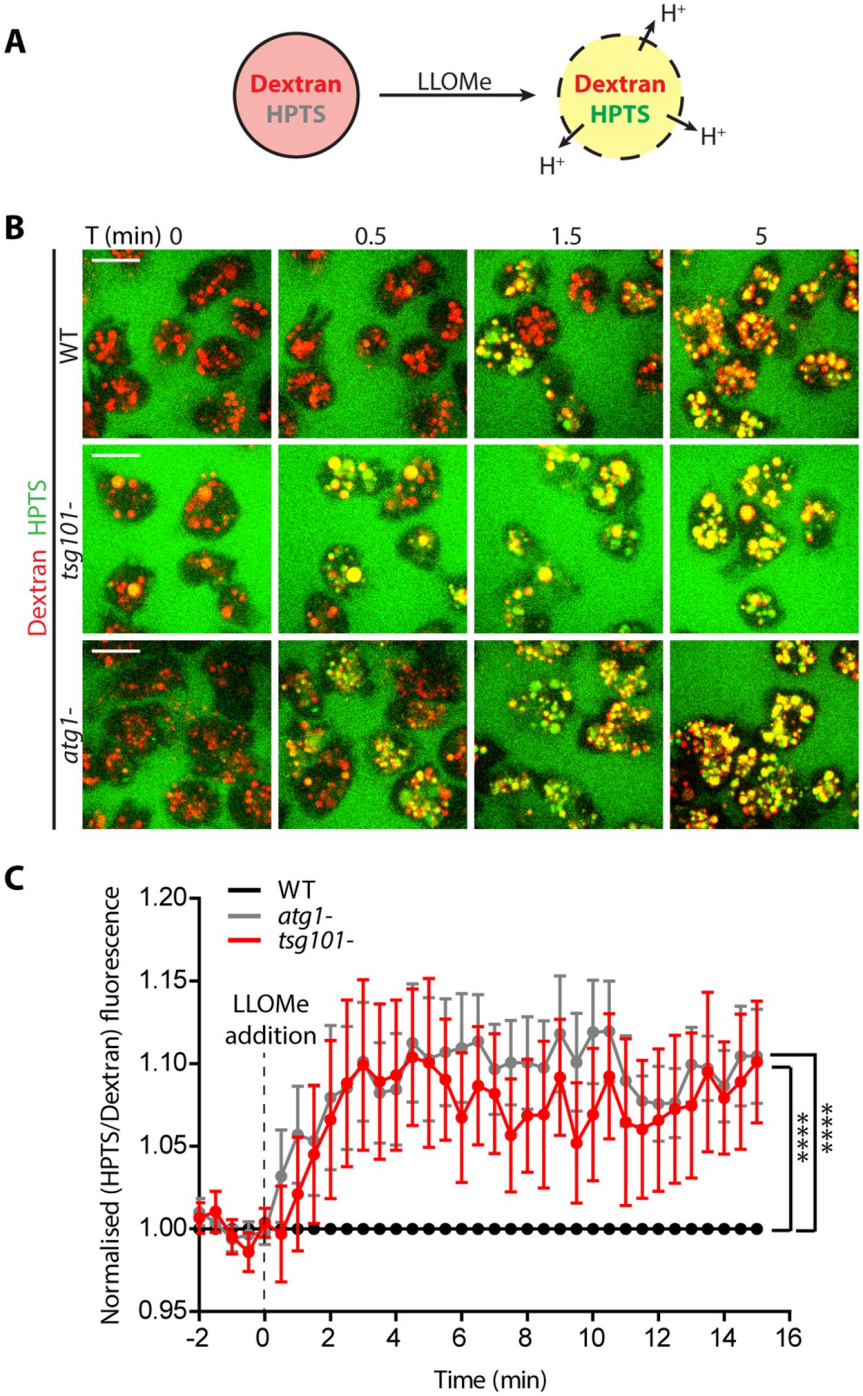
Increased leakage of lysosomes in ESCRT and autophagy mutants upon LLOMe treatment. (A) Schematic representation of the experiment shown in B. Cells were treated for at least 3 h with the Alexa Fluor 647 10 kDa dextran (red) to label all endosomes, together with the 0.5 KDa soluble pH indicator HPTS (green). (B) *D*. *discoideum* wt, *tsg101-* and *atg1-* were subjected to the experimental procedure depicted in A and monitored by time-lapse imaging (see also S7 Movie). Before addition of LLOMe, HPTS was quenched in acidic lysosomes, which therefore appeared in red. HPTS dequenching started after 30 sec in *tsg101-* and *atg1-* cells and after 1.5 min in wt cells. (C) Quantification of the time-lapse experiments shown in B. The ratio of the intensities of HPTS and Alexa Fluor 647 10 kDa dextran in sum projections is plotted as a function of time. The graph shows the mean and SEM of N = 10 independent experiments. Two-way ANOVA and post hoc Fisher’s LSD tests were performed.

To further dissect the functional contributions of the ESCRT-III and autophagy machineries to the repair of wounds inflicted by LLOMe, cells were incubated with a mixture of two fluid-phase markers: the 10 kDa pH-insensitive Alexa Fluor 647 dextran and the 0.5 kDa pH sensor 8-Hydroxypyrene-1,3,6-trisulfonic acid, trisodium salt (HPTS), which is quenched at pH < 6.5 (Fig 6A–6C and S8 Movie). Around 5 min after LLOMe addition, the HPTS fluorescence increased drastically and synchronously in the lysosomes of wt cells, indicating proton leakage from the compartments. In the autophagy mutant *atg1*-, which are defective in MCV/endomembrane repair [23], the fluorescence dequenching happened faster and was more pronounced, a sign of earlier and more extensive proton leakage. Interestingly, in *tsg101*- cells, the switch in fluorescence also happened earlier and more intensely, again suggesting as for the *atg1*- cells a defect in membrane repair in these mutants.

### Deficiencies in membrane repair lead to earlier escape of *M*. *marinum* from the MCV

To decipher the role of ESCRT during infection, cells lacking Tsg101 or the accessory proteins AlxA and the AlxA interactors Alg2a and Alg2b were infected and examined by EM. In wt cells, *alxA-* or *alg2a-/b-* mutants, the membrane of the MCV in close vicinity to the bacilli escaping the compartment was even and smooth (S4A, S4D–S4F Fig). However, in the *tsg101-* mutant, rough and “bubbling” membrane structures were observed (S4B and S4C Fig), suggesting cumulating membrane damage. In all cases, escaping bacteria were surrounded by the highly electron-dense material already described in Fig 1.

**Fig 7:**
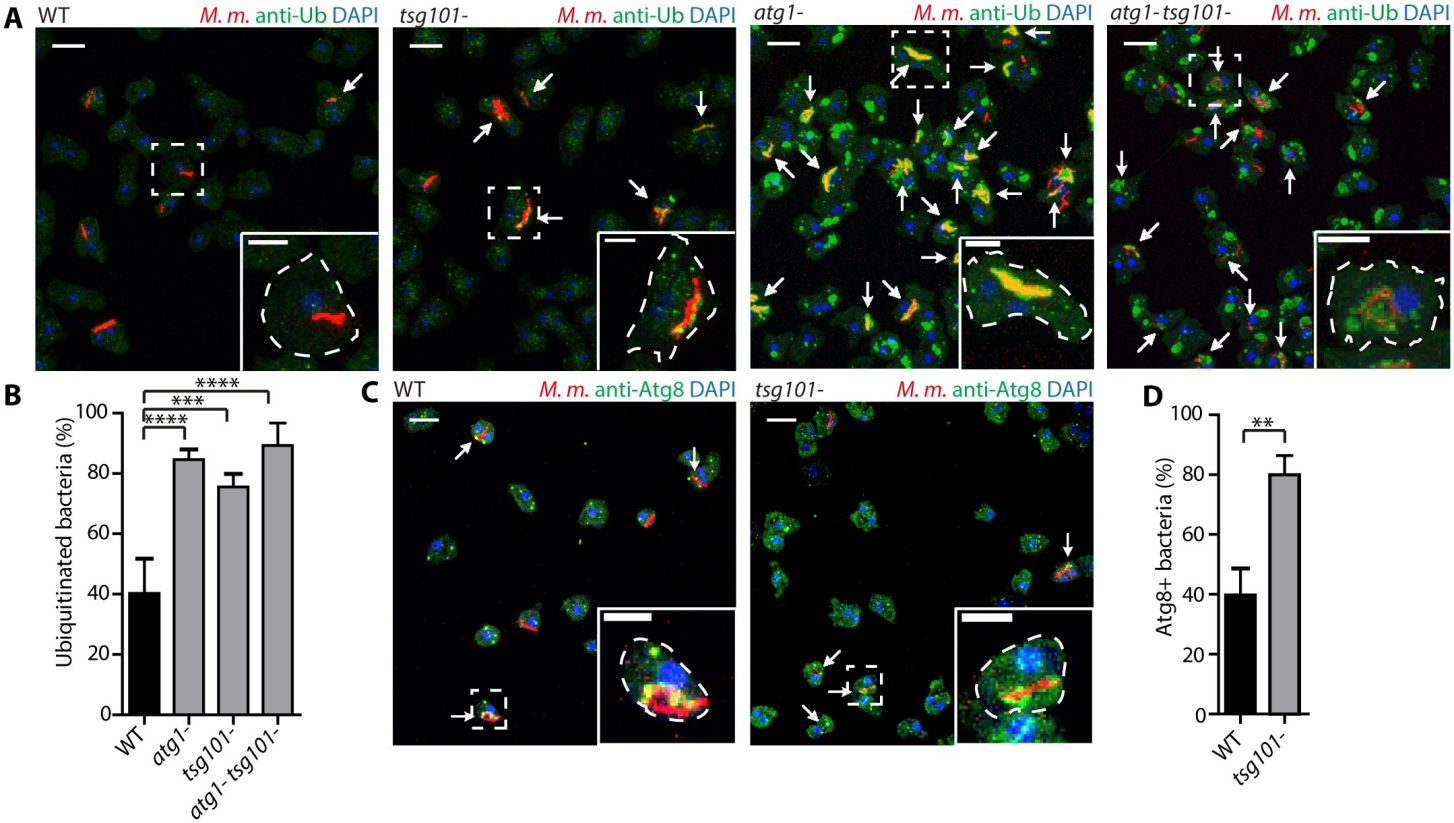
Deficiency in membrane repair leads to earlier escape of *M*. *marinum* from the MCV. (A) *D*. *discoideum* wt or mutant (*atg1*-, *tsg101*-, *atg1*- *tsg101*-) were infected with *M*. *marinum* and fixed for immunostaining at 8 hpi (*M*. *marinum* in red, ubiquitin in green, DAPI in blue). Arrows point to ubiquitinated bacteria. Scale bars, 10 μm and 5 μm for the insets. (B) Quantification of the proportion of ubiquitinated bacteria or bacterial microcolonies. The plot shows the mean and standard deviation (WT N = 6, n = 306; *atg1-* N = 3, n = 251; *tsg101*- N = 3, n = 93; *atg1*- *tsg101*- N = 3, n = 215). (C) Wt or *tsg101-* cells were infected with *M*. *marinum* and fixed for immunostaining at 8 hpi (*M*. *marinum* in red, Atg8 in green, DAPI in blue). Arrows point to bacteria decorated with Atg8. Scale bars, 10 μm and 5 μm for the insets. (D) Quantification of the proportion of bacteria or bacterial microcolonies decorated with Atg8. The plot shows the mean and standard deviation. (WT N = 3, n = 70; *tsg101-* N = 3, n = 63). Two-tailed *t*-tests (D) or two-way ANOVA and post hoc Fisher’s LSD tests were performed (B).

It was shown that in the *atg1-* mutant, *M*. *marinum* escapes earlier from the MCV, accumulates ubiquitin but proliferates more efficiently in a cytosol devoid of a bactericidal xenophagy pathway [23]. We reasoned that, if the ESCRT-III machinery were involved in repair of the MCV, then, in the *tsg101*- mutant, bacteria might access the cytosol and become ubiquitinated earlier. The percentage of ubiquitinated *M*. *marinum* at 8 hpi was significantly higher in the *tsg101-* (75.5 ± 4.3%) than in wt cells (40.3 ± 11.6%, Fig 7A and 7B, S5C Fig). In agreement with the increased ubiquitination of bacteria, *M*. *marinum* also colocalized more with Atg8 in the *tsg101*- (Fig 7C and 7D, S5D Fig). Although the percentage of ubiquitinated bacteria in *tsg101-* cells was close to that observed in the *atg1-* and *atg1- tsg101-* double mutants (84.6 ± 3.3% and 89.3 ± 7.5%, respectively), the extent of ubiquitin decoration on the bacteria was very different (Fig 7A and 7B, S5C Fig). Whereas in cells lacking Tsg101 ubiquitin formed foci or patches around *M*. *marinum*, in cells devoid of autophagy bacteria were more densely coated with ubiquitin (Fig 7A and S5C Fig). This accumulation is probably due to the fact that ubiquitinated bacteria cannot be targeted to autophagic degradation in the *atg1-* mutant, but autophagy is still functional in the *tsg101*- mutant.

Given that both ESCRT-III and autophagy are involved in the biogenesis of MVBs and autophagosomes, respectively, which rely at least partially on the recognition of ubiquitinated cargoes, we monitored the morphology of endosomes, as well as the levels of ubiquitination, in non-infected ESCRT and autophagy mutants (S5 Fig). In the *atg1-* and *atg1- tsg101-* mutants accumulation of high levels of ubiquitinated material was observed, in agreement with the inability of these mutants to degrade it by autophagy. In *tsg101*- cells, only a minor increase of ubiquitin was observed in endosomal compartments (S5A and S5B Fig), as already reported [50], which does not explain the more frequent and larger ubiquitin decorations around *M*. *marinum* in these cells (Fig 7A).

In yeast and mammallian cells devoid of some ESCRT proteins, ubiquitinated cargoes are not properly sorted into MVBs and accumulate on the limiting membrane [51]. Therefore, to confirm that the increase in ubiquitination observed during infection of the *tsg101*- mutant was due to MCV damage and bacteria access to the host cytosol, and not to failed endocytic cargo sorting, we monitored the colocalization of bacteria with GFP-tagged perilipin (Plin). Plin is a lipid droplet protein that binds the cell wall of *M*. *marinum* as soon as the bacteria access the cytosol [52]. Like in *atg1*- cells [52], recognition of cytosolic *M*. *marinum* by GFP-Plin was higher in *tsg101*- and *atg1*- *tsg101*- compared to wt cells (S5E and S5F Fig), confirming the earlier bacteria escape from the MCV in cells lacking a functional ESCRT machinery. Altogether, these results suggest that both Tsg101 and Atg1 trigger separate membrane repair pathways and restrict *M*. *marinum* access to the cytosol during infection.

Since we have shown that Tsg101 is not essential for ESCRT-III recruitment to the damaged MCV (Fig 2G and 2H), but has an important role in repairing the MCV and constraining bacteria escape (Fig 7 and S5 Fig), we wondered whether the accessory proteins AlxA and Alg2a/b, also known to recruit ESCRT-III, were involved in the repair of the MCV. In cells lacking Alg2a/b, the percentage of ubiquitinated *M*. *marinum* was comparable to that in its respective parental strain (43.3 ± 15.0% and 50.2 ± 11.4% respectively, S6A, S6B and S6E Fig) and, similarly, the degree of Atg8 colocalization with the bacteria remained lower in the *alg2a-/b-* mutant (54.70 ± 9.5%, S6C, S6D and S6F Fig). On the contrary, 81.8 ± 9.9% of bacteria were ubiquinated in cells devoid of AlxA, which correlated with a higher but not significant increase of Atg8 recruitment to the bacteria (72.7 ± 13.0%, S6A–S6F Fig). This suggests that AlxA, together with Tsg101 but not Alg2a/b, contributes to the ESCRT-III-mediated repair of the MCV.

### Impairment of ESCRT or autophagy has a distinct impact on *M*. *marinum* intracellular growth

**Fig 8:**
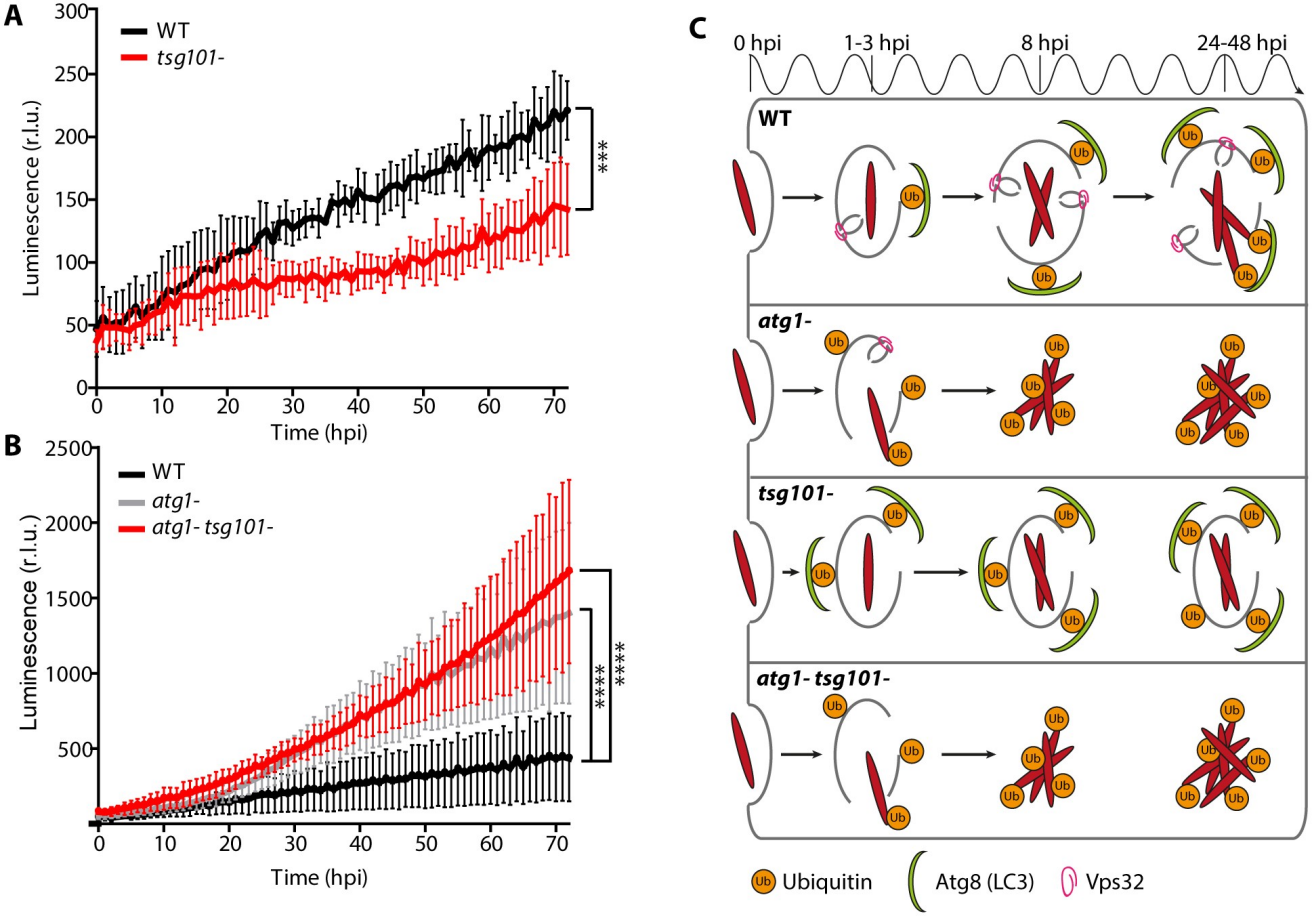
Impact of ESCRT and autophagy on mycobacterial growth. (A-B) Wt or mutant *D*. *discoideum* were infected with luminescent *M*. *marinum* and intracellular bacterial growth was monitored in a plate reader over 72 hpi. *M*. *marinum* growth was restricted in the *tsg101-* mutant, whereas it hyperproliferates in the *atg1-* and the double *atg1- tsg101-* mutant. Plots represent the mean and standard deviation of N = 3 independent experiments. Two-way ANOVA and post hoc Fisher’s LSD test were performed. (C) Model of autophagy and ESCRT-III involvement in MCV repair and restriction of *M*. *marinum* intracellular growth. In wt *D*. *discoideum*, *M*. *marinum* induces ESX-1-dependent injuries in the MCV membrane. This leads to the separate recruitment of the ESCRT-III and autophagy machineries to repair the damage, but nevertheless the bacteria are able to access the cytosol at later stages of infection (24–48 hpi). In the *atg1-* mutant, *M*. *marinum* access the cytosol earlier, despite the membrane repair exerted by ESCRT-III. Bacteria accumulate ubiquitin and proliferate more extensively in the cytosol devoid of a functional autophagy pathway. In the *tsg101-* mutant, membrane damage not repaired by ESCRT-III leads to an increase of ubiquitination and recruitment of the autophagy machinery, resulting in restriction of *M*. *marinum* growth. In a double *atg1- tsg101-* mutant, lack of both ESCRT-III and autophagic membrane repairs leads to an earlier access of bacteria to the cytosol, which accumulate ubiquitin and hyperproliferate as in the single *atg1-* mutant.

To study how the ESCRT pathway may impact the outcome of *M*. *marinum* infection, the ESCRT mutants were infected with luminescent *M*. *marinum* [53] and intracellular bacterial growth monitored [54] (Fig 8A and 8B, S6G and S6H Fig). *M*. *marinum* luminescence increased around 5- fold in wt *D*. *discoideum* in the course of 72 h, reflecting sustained intracellular growth. In the *atg1-* mutant, since bacteria escape earlier to a cytosol that is devoid of xenophagic defense, *M*. *marinum* grew better (Fig 8B), as already described [23]. *M*. *marinum* proliferation in both ESCRT mutants *alxA-* and *alg2a-/b-* was similar to that in the wt (S6G and S6H Fig), suggesting no crucial involvement of these proteins in the infection course. Importantly, loss of Tsg101 significantly suppressed *M*. *marinum* growth compared to wt cells (Fig 8A). Interestingly, in the double mutant *atg1- tsg101-*, bacterial luminescence increased substantially, reaching similar levels as in the single *atg1*- mutant. Therefore, a functional autophagy pathway is necessary to control bacterial burden in the *tsg101*- mutant indicating that without ESCRT-III-mediated MCV repair the bacteria become more accessible to degradation by xenophagy. Consistent with its decreased ability to access the cytosol, *M*. *marinum* ΔRD1 grew very poorly in *D*. *discoideum* wt cells, and this attenuated growth was not improved in the *atg1-*, *tsg101-* and *atg1- tsg101-* mutants. (S7 Fig). Taken together with the previous results on ubiquitination and Atg8 recruitment (Fig 7 and S6 Fig), we conclude that Tsg101 and AlxA but not Alg2a/b participate in the ESCRT-III-mediated repair of the MCV damage and thus absence of these proteins enables an earlier escape of *M*. *marinum* to the cytosol. In the case of *tsg101-*, this leads to the early recruitment of the autophagy machinery, which restricts bacterial mass.

## Discussion

While most intracellular bacterial pathogens reside in a vesicular compartment where they exploit the host resources, a few bacteria have adapted to translocate to the host cytosol. The dynamics of escape to the cytosol varies depending on the pathogen. For instance, while *Shigella* and *Listeria* trigger an early escape, *Salmonella* and mycobacteria program a partial and/or delayed escape [10]. This is the case of *M*. *marinum*, which disrupts the MCV thanks to a combination of the membranolytic activity of ESAT-6, a small bacterial peptide secreted by the ESX-1 system [55], and the action of the mycobacterial cell wall PDIMs [6, 7]. Perforation of the MCV implies first the leakage of host and bacterial factors contained in its lumen and, eventually, bacteria access to nutrients in the cytosol, which must be sensed and restricted by the host. 3D EM inspection of infected *D*. *discoideum* cells revealed a very complex interface between *M*. *marinum* and the host cytosol at the site of MCV rupture (Fig 1), suggesting a dynamic and complex interplay between bacterial and host factors. Here, we show that the two highly conserved ESCRT-III and autophagy pathways contribute to the repair of the MCV membrane, delaying the escape of *M*. *marinum* to the cytosol.

Like its mammalian homologs [26–28, 42, 43], the *D*. *discoideum* ESCRT proteins Tsg101, Vps32 and Vps4 localized to injuries both at the plasma membrane and endomembranes upon damage by distinct chemical agents such as digitonin and LLOMe (Fig 4A and 4B). Importantly, *M*. *marinum* infection also leads to the appearance of Tsg101, Vps32 and Vps4 foci, patches or rings in the vicinity of the MCV (Fig 2). These structures were significantly less abundant upon infection with *M*. *marinum* ΔRD1, an attenuated mutant that produces PDIMs (S1 Fig) but lacks the ESX-1 secretion system. Although we cannot exclude a direct role of the ESX-1 secretion system or of another secreted product in the recruitment of the ESCRT machinery, this result is consistent with the reduced capacity of *M*. *marinum* ΔRD1 to induce damage at the MCV. In agreement with its ability to form packed spiral polymers on membranes, upon both sterile injuries and infection, GFP-Vps32 structures were generally larger and longer-lived than the Vps4-GFP ones. Consistently, large ring-like structures were observed exclusively with GFP-Vps32 (Fig 2E). Time-lapse microscopy revealed that GFP-Vps32 was recruited from the cytosolic pool, likely polymerized at wounds of the MCV, and remained associated with the MCV for several minutes (Fig 3C).

The wounds inflicted by membrane disrupting agents such as LLOMe (less than 5 nm [56]) may be of comparable size to the ones caused by the mycobacterial membranolytic peptide ESAT-6 (4.5 nm [8]) and thus lead similarly to the recruitment of the ESCRT-III repair machinery. However, the sustained insults and cumulative lesions inflicted by *M*. *marinum* [57] likely results from the continued damage caused by ESAT-6 and PDIMs [6, 48]. Together, they probably generate heterogeneous and expanding wounds that are harder to resolve. This may explain why the recruitment of GFP-Vps32 to sterile damage depends strictly on Tsg101 (Fig 5C and 5D), whereas during *M*. *marinum* infection this dependency is partial (Fig 2G and 2H), because other ESCRT recruiting pathways likely act simultaneously. In addition, we propose that cumulative damage by *M*. *marinum* would eventually overwhelm membrane repair by ESCRT-III and result in recruitment of autophagy, as previously suggested for endosomal damage caused by LLOMe [28, 29]. In the case of sterile damage to lysosomes, autophagy may end up degrading the severely injured compartments by lysophagy [28]. Regarding damage to the MCV, we propose that autophagy plays two distinct roles. First, in membrane repair, where autophagic membranes would somehow patch/seal the damaged MCV and contain the bacteria in the compartment, as proposed also during *Salmonella* infection [25]. The second one implies the total engulfment of the MCV for the degradation of its content, similarly to the already described role of autophagy in canonical lysophagy. These two paths would generate compartments/environments that are either restrictive or even bactericidal for the pathogen.

Upon digitonin treatment, GFP-Vps32 colocalized with Annexin V-labelled regions of the plasma membrane that were subsequently shed in the medium (Fig 5B). This suggests that plasma membrane repair in *D*. *discoideum* might be achieved by ESCRT-III-dependent budding and scission of the wound, as in mammalian cells [26, 27]. A similar repair mechanism, in this case by budding the injury towards the lumen, has also been described for the nuclear envelope [43]. We propose that the same process may operate at the MCV, which would partially explain the presence of abundant membranous material in the lumen of the MCV and the putative invagination of the MCV membrane remnants, as observed by EM and live microscopy (Fig 1 and S2A–S2C Fig).

*M*. *marinum* that accesses the host cytosol becomes ubiquitinated [23] and coated by Plin [52]. We used ubiquitination and recruitment of GFP-Plin as a proxy to monitor bacterial escape from the MCV in cells lacking Tsg101. Similar to the high ubiquitination previously shown to occur in amoebae deficient for autophagy [23], large ubiquitin patches were observed at the sites of MCV rupture in *tsg101*- cells (Fig 7A and 7B). Consistently, the percentage of bacteria coated by Plin was higher in this ESCRT mutant (S5E and S5F Fig). These results are consistent with the large ubiquitin patches observed on *M*. *smegmatis* in macrophages [30] and with the increased cytosolic bacterial spread observed during *L*. *monocytogenes* infection in S2 cells [41] when ESCRT genes are downregulated.

Several reports have recently described the participation of ESCRT proteins during micro- and macro-autophagy (reviewed in [58]). However, during endolysosomal membrane repair, ESCRT-III has been shown to operate independently of lysophagy [28, 29]. In agreement, upon treatment with LLOMe, we also observe GFP-Vps32 and Vps4-GFP recruitment preceding GFP-Atg8. In the case of MCV rupture, we suggest that ESCRT-III and autophagy work in parallel to repair the damaged membrane. This function of autophagy somehow supplying membrane to seal/patch the damaged MCV explains the early escape of *M*. *marinum* to the cytosol in the *D*. *discoideum atg1-* mutant and has also been described during *Salmonella* infection [25]. In addition, it has been shown that ubiquitin serves as an “eat-me” signal that targets cytosolic bacteria to autophagic degradation [2]. This second and distinct function probably implies the total engulfment of the (damaged) MCV, similarly to the role of autophagy in canonical lysophagy [28, 29]. Consistent with this, the accumulation of ubiquitin around *M*. *marinum* in *tsg101-* cells correlated with a proportional increase of Atg8 decoration on the bacteria (Fig 7C and 7D), and with a decreased bacterial load (Fig 8A), contrary to what has been described in RAW macrophages infected with the non-pathogenic, vacuolar *M*. *smegmatis*, in which depletion of Tsg101 led to bacteria hyperproliferation [30]. Importantly, elimination of the autophagic function in both wt or *tsg101*- mutant cells led to a significant increase of intracellular mycobacterial growth, thus suggesting that bacterial restriction observed in the single *tsg101*- mutant is due to autophagic restriction (Fig 8B). However, it is important to note that the 20-fold increase of bacterial mass observed in the absence of autophagy is notably reduced compared to the 512-fold that is obtained over the same period of time during exponential growth in 7H9 medium. The identity of the additional, autophagy-independent, growth-restriction pathways, in the MCV or in the cytosol, are not fully elucidated, but will likely include access to nutrients and other bacteria-limiting mechanisms such as ROS production and anti-bacterial activities [1]. These anti-bacterial machineries must also restrict the growth of *M*. *marinum* ΔRD1, either in the MCV or in the cytosol for the small percentage (roughly 5%) that may escape (S7 Fig).

Similarly to the substrates of ubiquitination, the cues and signals that recruit ESCRT-III to damage in *D*. *discoideum* are still to be identified. The appearance of ESCRT-III components before ubiquitinated material can be detected at the site of lysosome disruptions [28] speaks for a ubiquitin-independent mechanism, although it cannot be excluded that ubiquitin might participate in a subsequent reinforcement loop to recruit ESCRT-III. In mammalian cells, several reports have suggested that influx of extracellular Ca^2+^ through the plasma membrane or efflux through endolysosomal membranes are essential for the positioning of ESCRT-III to the site of the injury [26–28]. The local increase of intracellular Ca^2+^ at the wound site might be recognized directly by ALIX [26], a multifunctional protein involved in cargo protein sorting into intralumenal vesicles (reviewed in [59]), thereby bypassing the need for ESCRT-0, -I and -II, and recruiting ESCRT-III by direct protein-protein interactions. Alternatively, Ca^2+^ has been proposed to be sensed by ALG2, an ALIX-interacting protein with a penta EF-hand domain, which could promote the accumulation of ALIX, ESCRT-III and the Vps4 complex at the damage site [27, 28]. In the presence of EGTA and BAPTA-AM to chelate Ca^2+^, GFP-Vps32 relocation to the plasma membrane or lysosomal injuries was not impaired (Fig 5E and 5F). Consistent with this result, Ca^2+^ seemed also to be dispensable during MCV repair, since knockout of either *alxA* or *alg2a/b* did not impact intracellular *M*. *marinum* growth (S5G and S5H Fig). Altogether, our results suggest that the MCV damage caused by the *M*. *marinum* ESX-1 secretion system is repaired very robustly and in a multilayered response by both the ESCRT-III and the autophagy pathways of *D*. *discoideum* in a Tsg101-dependent and apparently Ca^2+^-independent manner. The ability of the ESCRT-III to repair membranes injured by various biological and chemical insults strongly suggests that this is a generic mechanism that operates upon infection by other intracellular pathogens. This is in agreement with a recent report showing ESCRT-III proteins at vacuoles containing *C*. *burnetii* [29] and will need to be addressed in the case of other membrane-damaging bacteria such as *Salmonella*, *Shigella* and *Listeria*. In addition, the high conservation of the ESCRT machinery identifies this pathway as a novel potential therapeutic target to fight against bacterial infection in humans.

## Materials and methods

### *D*. *discoideum* strains, culture and plasmids

*D*. *discoideum* strains and plasmids are summarized in S1 Table. *D*. *discoideum* Ax2(Ka) was axenically cultured at 22°C in Hl5c medium (Formedium) supplemented with 100 U mL^−1^ of penicillin and 100 μg mL^−1^ of streptomycin (Invitrogen). *D*. *discoideum* JH10 was cultured similarly as Ax2(Ka), with the addition of 100 g mL-1 of Thymidine (Sigma). Cells were transformed by electroporation and selected with the relevant antibiotic. Hygromycin, G418 and blasticidin were used at a concentration of 50, 5 and 5 μg mL^−1^, respectively. *D*. *discoideum* Ax2(Ka) *tsg101-* and *atg1- tsg101-* were obtained by transformation with the pSP72 KO vector kindly provided by Dr. L. Aubry [60]. JH10 *alxA-* and JH10 *alg2a-/b-* mutants were kindly provided by Dr. L. Aubrey [61, 62]. Ax2(Ka) cells expressing GFP-Atg8 and AmtA-mCherry were described in [23] and [52], respectively. Ax2(Ka) cells expressing GFP-Vps32, Vps4-GFP, GFP-Tsg101 and GFP-Atg8 and RFP-Vps32 were generated by transformation with the vectors described in S1 Table. Cells expressing GFP-Plin were obtained upon transformation with the pDNeoGFP-Plin construct described in [63].

### Mycobacteria strains, culture and plasmids

*M*. *marinum* strains and plasmids are summarized in S1 Table. *M*. *marinum* (M strain) wt and ΔRD1 were kindly provided by Dr. L. Ramakhrisnan [23]. Mycobacteria were cultured in 7H9 (Difco) supplemented with glycerol 0.2% (v/v) (Biosciences), Tween-20 0.05% (v/v) (Sigma) and OADC 10% (v/v) (Middlebrock). mCherry-expressing bacteria were obtained by transformation with pCherry10, which was kindly obtained from Dr. L. Ramakrishnan [64], and cultured in the presence of 50 μg/mL^-1^ hygromycin (Labforce). Luminiscent bacteria harbored the pMV306::*lux* plasmid [53] and were cultured in presence of 50 μg mL^-1^ kanamycin (AppliChem).

### Infection assay

Infections were performed as previously described [65]. In brief, *M*. *marinum* bacteria were washed in Hl5c and centrifuged onto adherent *D*. *discoideum* cells. After additional 20–30 min of incubation, extracellular bacteria were washed off and the infected cells resuspended in Hl5c containing 5 U mL^-1^ of penicillin and 5 μg mL^-1^ of streptomycin (Invitrogen). Infections were performed at a multiplicity of infection (MOI) of 10 for *M*. *marinum* wt in *D*. *discoideum* wt. In order to correct for slight differences in phagocytic uptake, infections with *atg1-* and *atg1- tsg101- D*. *discoideum* mutants were performed at MOI 5, and with *tsg101-* at MOI 20. For experiments with *M*. *marinum* ΔRD1 the MOI used was always twice the MOI of *M*. *marinum* wt. For live microscopy, mCherry expressing or unlabeled bacteria were used. To monitor bacteria intracellular growth in *D*. *discoideum*, luciferase-expressing *M*. *marinum* [23, 54] were used.

### Intracellular growth measurement

Growth of luminescent bacteria was measured as described previously [23, 54]. Briefly, dilutions of 0.5–2.0 × 10^5^ *D*. *discoideum* cells infected with *M*. *marinum* pMV306::*lux* were plated on a non-treated, white F96 MicroWell plate (Nunc) and covered with a gas permeable moisture barrier seal (Bioconcept). Luminescence was measured for 72 h at 1 h intervals with a Synergy Mx Monochromator-Based Multi-Mode Microplate Reader (Biotek). The temperature was kept constant at 25°C.

### Live fluorescence microscopy

Infected or uninfected *D*. *discoideum* were plated in 35 mm Glass Bottom Dishes (MatTek) or in 4-wells μ-slides (Ibidi). To induce damage in *D*. *discoideum* membranes, cells were incubated with 2.5–5 μM of digitonin (Sigma), 5–6.25 mM of LLOMe (Bachem), or 200 μM of GPN. These treatments were started immediately before live imaging, except in the case of LLOMe, for which a 10-fold concentrated solution was added once imaging had just started. To visualize the lumen of endocytic compartments, 0.5 mg mL^-1^ of 70 KDa TRITC-Dextran or 10–15 μg mL^-1^ of 10 kDa Alexa Fluor 647 Dextran (Molecular Probes) were added at least 3 h prior to visualization of the sample. To detect neutralization of endocytic vesicles, 0.2 mM of 524 Da HPTS (Molecular Probes) was added at least 3 h prior to the visualization of the sample. To detect plasma membrane damage, 5 μM of Annexin V Alexa Fluor 594 conjugate (Molecular Probles) was added to cells in the presence of 2.5 mM Ca^2+^. To chelate Ca^2+^, 5 mM of EGTA (Fluka) or 5 mM of EGTA and 250 μM of BAPTA-AM (Sigma) were added at least 20 min before imaging. Bacteria not expressing a fluorescent reporter were stained with 12.5 μM of Vybrant DyeCycle Ruby stain (Molecular Probes) directly on the μ-dish prior to microscopic inspection. Live microscopy was performed with a Spinning Disc Confocal Microscope [Intelligent Imaging Innovations Marianas SDC mounted on an inverted microscope (Leica DMIRE2)] with a glycerin 63x or an oil 100x objective. Generally, z-stacks from 3 to 10 slices of 1 μm or 1.5 μm were acquired. For time-lapse experiments, images were acquired from every 15 s to several min. Image analysis was performed using ImageJ.

### Cell fixation and immunofluorescence stainings

Samples were fixed in ultracold methanol as already described [66]. Briefly, *D*. *discoideum* cells on coverslips were quickly fixed by immersion in -85°C methanol using a Freezing Dewar (FH Cryotec, Instrumentenbedarf Kryoelektronenmikroskopie). Subsequent immunofluorescence staining was performed as described [66]. Rabbit anti-Atg8 was previously described [23], anti-p80 [67] was purchased from the Geneva Antibody Facility (http://www.unige.ch/antibodies), the anti-Ub (FK2) monoclonal antibody was from Enzo Life Sciences [23]. Nuclei were stained with DAPI (Molecular Probes). Cells were embedded using ProlongGold antifade (Molecular Probes). Images were acquired with a LSM700 or LSM780 microscope (Zeiss) using an oil 63x objective. Image analysis was performed using ImageJ.

### Transmission Electron Microscopy (TEM)

Sample preparation for TEM was performed as described in [68]. Briefly, *D*. *discoideum* cells were fixed in a 6 cm dish in 2% (w/v) glutaraldehyde in Hl5c for 1 h and stained with 2% (w/v) OsO_4_ in imidazole buffer 0.1 M for 30 min. Cells were detached with a cell scraper and washed 3 times with PBS. Subsequent sample preparation was performed at the Pôle Facultaire de Microscopie Ultrastructurale (University of Geneva). Samples were incubated in 2% (v/v) of Milloning buffer and rinsed with distilled water. Then, they were incubated in 0.25% (w/v) uranyl acetate overnight and rinsed with distilled water. Samples were dehydrated using increasing concentrations of ethanol, then in propylene oxide for 10 min and finally embedded in 50% Epon-propylene oxide for 1h, followed by incubation overnight in pure Epon. Samples were embedded in 2% agar for subsequent sectioning in an ultramicrotome and placed on TEM grids. Finally, sections were visualized in a Tecnai 20 electron microscope (FEI Company, Eindhoven, The Netherlands).

### Focus Ion Beam Scanning Electron Microscopy (FIB-SEM)

Initial sample preparation was performed similarly as for TEM and sent to the Pôle Facultaire de Microscopie Ultrastructurale (University of Geneva). Subsequent contrast enhancement, dehydration and resin embedding was performed as described in [69]. Samples were visualized in a Helios DualBeam NanoLab 660 SEM (Fei Company, Eindhoven, The Netherlands). 3 D reconstitutions were performed using the LimeSeg plugin from ImageJ.

### Thin Layer Chromatography (TLC)

For the analysis of the lipid composition, *M*. *marinum* strains were grown in suspension in 25 ml 7H9 supplemented with ADC and 0.05% Tyloxapol. At an OD_600_ of 1 the bacteria were harvested, washed in PBS and resuspended in 1 ml of water. The non-polar lipid fraction was extracted using Bligh and Dyer [70] and separated on a TLC plate using petroleum ether/ethyl acetate (98:2) as a solvent system. The *M*. *marinum tesA* mutant inhibited in the synthesis of both, PDIMs and PGLs [71], served as a negative control. Lipids were visualised after charring in a solution containing MnCl_2_, methanol and sulfuric acid and heating for five minutes at 150 °C. Purified PDIMs of *M*. *tuberculosis* (Biodefense and Emerging Infections Research (BEI) resources (NIAID, NIH) and glyceryl trioleate (Fluka) were used to identify bands on the TLC.

### Statistics

Microscopy images were analysed using ImageJ. Experiments in Figs 2B, 2C, 2D, 2H, 7B and 7D; S5F, S6B and S6D Figs were quantified manually. Experiments in Fig 6C were quantified automatically using ImageJ macros. Briefly, endosomes were identified using the Far-Red Dextran signal and fluorescence intensities of the HTPS and Far-Red Dextran for each endosome were measured. The ratio of the mean intensity of each channel per time-point was calculated and normalized to the baseline (5 first time-points prior to LLOMe treatment) and then to the wt for all the time-points. Experiments in Figs 4 and 5 were quantified using MetaXpress software. All graphs were plotted and statistical tests were performed using Prism. In all plots, the mean and standard deviation are shown, unless explicitly mentioned. Two-tailed t-test, ANOVA or Two-way ANOVA was used. Post hoc Fisher’s LSD test were performed when necessary (n.s: non-significant, *: p-value < 0.05, **: p-value < 0.01, ***: p-value < 0.001, ****: p-value < 0.0001).

## Supporting information

S1 Table List of strains and plasmids. (DOCX)

S1 Fig *M*. *marinum* wt and *M*. *marinum* ΔRD1 synthesize similar levels of PDIMs. Lipids from *M*. *marinum* wt and *M*. *marinum* ΔRD1 were analysed by Thin Layer Chromatography (TLC). Presence of PDIMs was detected in both strains, in contrast to the PDIM defective mutant *M*. *marinum* ΔTesA used as a control [71]. The asterisk marks a lipid of unknown nature.
(TIF)

S2 Fig Membrane invaginations at the MCV. (A) High magnification inset of the picture shown in Fig 3B 31 hpi, to highlight the topology of GFP-Vps32 recruitment (yellow arrow) at the damaged MCV (labelled with AmtA-mCherry). The membrane of the compartment seems to invaginate towards the lumen of the MCV (white arrow). (B-I). *D*. *discoideum* cells were infected with *M*. *marinum* (“*M*.*m*.” labelled in red) and fixed for TEM to visualize the ultrastructure of the MCV. (B-C) Membrane invaginations at the MCV covered by electron-dense cytosolic material are highlighted with orange arrows. Image in (C) is a high magnification of Fig 1E in [23]. Scale bars, 1 μm.
(TIF)

S3 Fig Various membrane damaging agents induce ESCRT proteins recruitment to the site of rupture. (A) *D*. *discoideum* cells co-expressing GFP-Atg8 and RFP-Vps32 were treated with LLOMe and monitored by time-lapse microscopy. Circular RFP-Vps32 structures were observed several minutes before GFP-Atg8 became apparent (see also S6 Movie). (B) *D*. *discoideum* expressing GFP-Vps32 or Vps4-GFP were treated with digitonin, GPN, LLOMe, or medium (control) and visualized over time. Still images show representative cells in phase-contrast and fluorescence at 0, 5, 10 and 15 min after treatment. On the right, magnification of one of the images per treatment. Red arrows point to GFP-Vps32 and Vps4-GFP structures at the sites of damage. Scale bars 10 μm. *D*. *discoideum* expressing GFP-Vps32 were incubated with TRITC-Dextran (red) (C) or Alexa Fluor 647 Dextran (red) (D) for at least 3 h to label all endosomes, treated with LLOMe or GPN, respectively, and monitored by time-lapse microscopy. Kymographs generated by a repeated linescan through a representative cell show the sustained association of GFP-Vps32 structures with the lysosomes and endosomes (black and white arrows). In D, the compound was added immediately before imaging started.
(TIF)

S4 Fig Ultrastructural appearance of the escape site of *M*. *marinum* in several ESCRT mutants. *D*. *discoideum* cells were infected with *M*. *marinum* and fixed for TEM at 24 hpi. *M*. *marinum* (“*M*.*m*.” labelled in red) accessed the cytosol in wt (A and D), *tsg101-* (B and C), *alix-* (E) and *alg2a-/b-* (F). Sites of membrane disruption are highlighted with blue arrows. (C) High magnification inset of the region of interest in (B). Scale bars, 1 μm.
(TIF)

S5 Fig Ubiquitination and GFP-Plin recruitment as readout of *M*. *marinum* cytosolic access. (A-B) *D*. *discoideum* wt or mutant (*atg1-*, *tsg101-* or *atg1- tsg101-*) were fixed for immunofluorescence. Post-lysosomes were labelled with p80 (green), nuclei were labelled with DAPI (blue), ubiquitin was in green and Atg8 in red. (C-D) *D*. *discoideum* wt or mutant (*atg1-*, *tsg101-*, or *atg1-tsg101-*) were infected with *M*. *marinum* (blue) and fixed for immunofluorescence. Both ubiquitinin and Atg8 (green) decorated the bacteria when the MCV (p80, red) was disrupted. Scale bars 1 μm. (E) *D*. *discoideum* wt or mutant (*atg1-*, *tsg101-*, or *atg1-tsg101-*) expressing GFP-Plin (green) were infected with *M*. *marinum* for live microscopy. All mutants showed an increase of GFP-Plin recruitment on the bacteria (red). (F) Proportion of the *M*. *marinum* bacteria or microcolonies decorated with GFP-Plin. The plot shows the mean and standard deviation (WT N = 4, n = 139; *atg1-* N = 3, n = 148; *tsg101-* N = 3, n = 98; *atg1- tsg101-* N = 3, n = 191). ANOVA and post hoc Fisher’s LSD test were performed.
(TIF)

S6 Fig AlxA and Alg2a/b do not impact *M*. *marinum* intracellular replication. (A) *D*. *discoideum* wt or mutant (*alxA-* or *alg2-/b-*) were infected with *M*. *marinum* and fixed for immunostaining at 8 hpi (*M*. *marinum* in red, ubiquitin in green, DAPI in blue). Arrows point to ubiquitinated bacteria. Scale bars, 10 μm and 5 μm for the insets. (B) Quantification of the proportion of ubiquitinated bacteria or bacterial microcolonies. The plot shows the mean and standard deviation [WT (JH10) N = 4, n = 144; *alxA-* (JH10) N = 3, n = 134; *alg2a-/b-* (JH10) N = 3, n = 266]. (C) *D*. *discoideum* wt or mutant (*alxA-* or *alg2-/b-*) were infected with *M*. *marinum* and fixed for immunostaining at 8 hpi (*M*. *marinum* in red, Atg8 in green, DAPI in blue). Arrows point to bacteria decorated with Atg8. Scale bars, 10 μm and 5 μm for the insets. (D) Quantification of the proportion of bacteria or bacterial microcolonies decorated with Atg8. The plot shows the mean and standard deviation [WT (JH10) N = 4, n = 275; *alxA-* (JH10) N = 4, n = 170; *alg2a-/b-* (JH10) N = 4, n = 448]. ANOVA and post hoc Fisher’s LSD test were performed. (E-F) *D*. *discoideum* wt or mutant (*alxA-* or *alg2-/b-*) were infected with *M*. *marinum* (blue) and fixed for immunofluorescence. Both ubiquitinin and Atg8 (green) decorate the bacteria when the MCV (p80, red) was disrupted. Scale bars, 1 μm. (G-H) *D*. *discoideum* wt or mutant (*alxA-* or *alg2-/b-*) were infected with luminescent *M*. *marinum* and intracellular bacterial growth was monitored in a plate reader over 72 hpi. There was no significant difference between *M*. *marinum* growth in wt and mutants. Plots represent the mean and standard deviation of N = 3 independent experiments. Two-way ANOVA and post hoc Fisher’s LSD test were performed.
(TIF)

S7 Fig *M*. *marinum* ΔRD1 growth is severally impaired in *D*. *discoideum*. *D*. *discoideum* wt or mutant (*atg1-*, *tsg101-* or *atg1-tsg101-*) were infected with luminescent *M*. *marinum* (wt or ΔRD1) and intracellular bacterial growth was monitored in a plate reader over 72 hpi. In all D. *discoideum* mutants tested, *M*. *marinum* ΔRD1 growth was significantly attenuated compared to *M*. *marinum* wt in a *D*. *discoideum* wt host. Plots represent the mean and standard deviation of N = 3 independent experiments. Two-way ANOVA and post hoc Fisher’s LSD test were performed.
(TIF)

S1 Movie The ultrastructure of the MCV at the site of *M*. *marinum* escape. FIB-SEM stack and corresponding 3D reconstruction of the damaged MCV shown in Fig 1D. “*M*.*m*.”, *Mycobacterium marinum* (in dark blue); “MCV”, *Mycobacterium*-containing vacuole (in light blue); electron-dense material surrounding the cytosolic bacteria, in yellow.
(MP4)

S2 Movie GFP-Vps32 forms rings in the vicinity of *M*. *marinum*. Time-lapse of a *D*. *discoideum* cell expressing GFP-Vps32 and infected with *M*. *marinum* wt (red). Arrows show GFP-Vps32 rings which formed and seemed to move along the bacterium. Scale bar, 10 μm.
(MP4)

S3 Movie Dynamics of GFP-Vps32 recruitment in the vicinity of the MCV. Time-lapse of a *D*. *discoideum* cell expressing GFP-Vps32 and AmtA-mCherry and infected with *M*. *marinum* wt (red). Scale bars, 10 μm.
(MP4)

S4 Movie Localisation of ESCRT and autophagy components upon digitonin treatment. Time-lapse imaging of *D*. *discoideum* cells expressing GFP-Tsg101, GFP-Vps32, Vps4-GFP or GFP-Atg8 upon digitonin treatment. Scale bar, 10 μm.
(MP4)

S5 Movie Localisation of ESCRT and autophagy components upon LLOMe treatment. Time-lapse imaging of *D*. *discoideum* cells expressing GFP-Tsg101, GFP-Vps32, Vps4-GFP or GFP-Atg8 upon LLOMe treatment. Cells were incubated with fluorescent dextran (red) for at least 3 h prior to the treatment. Scale bar, 10 μm.
(MP4)

S6 Movie Different temporal recruitment of GFP-Atg8 and RFP-Vps32 on damaged lysosomes. Time-lapse imaging of *D*. *discoideum* cells co-expressing GFP-Atg8 and RFP-Vps32 upon LLOMe treatment. Scale bar, 10 μm.
(MP4)

S7 Movie Annexin V labels membrane damage upon digitonin treatment. Time-lapse imaging of *D*. *discoideum* cells that were incubated with Annexin V Alexa Fluor 594 conjugate in the presence of Ca^2+^ and then treated with digitonin. Arrowheads point to the site of membrane disruption. Scale bar, 10 μm.
(MP4)

S8 Movie Lysosomal leakage upon LLOMe treatment. Time-lapse imaging of *D*. *discoideum* wt, *tsg101-* and *atg1-* that were incubated with the 10 kDa Alexa Fluor 647 dextran (red) and the 0.5 kDa soluble pH indicator HPTS (green) and then treated with LLOMe. Scale bar, 10 μm.
(MP4)
