# Supplementary material for: The ESCRT and autophagy machineries cooperate to repair ESX-1-dependent damage at the Mycobacterium-containing vacuole but have opposite impact on containing the infection
Source: PLoS Pathog. 2018 Dec 31;14(12):e1007501. doi: 10.1371/journal.ppat.1007501 (PMC6329560; doi:10.1371/journal.ppat.1007501)
Supplement: S1 Table — (DOCX) [file ppat.1007501.s001.docx]

**Supplementary information**

**S1 Table. List of strains and plasmids**

| **Strain/Plasmid** | **Relevant characteristics** | **Source/Reference** |
| --- | --- | --- |
| *D. discoideum* | | |
| Ax2(Ka) | wt |  |
| JH10 | wt |  |
| Ax2(Ka) *atg1*- | KO | [1] |
| Ax2(Ka) *tsg101*- | KO | This study |
| Ax2(Ka) *atg1- tsg101-* | KO | This study |
| JH10 *alxA-* | KO | [2] |
| JH10 *alg2a/b-* | KO | [3] |
| *M. marinum* | | |
| M strain | wt | L. Ramakrishnan (Washington University) |
| ∆RD1 | KO | L. Ramakrishnan (Washington University) |
| *D. discoideum* plasmids | | |
| pDM317 |  | [4] |
| pDM323 |  | [1] |
| pJSK500 | GFP-Atg8a | [5] |
| GFP-Tsg101 | *tsg101* cDNA (DDB_G0286797) in pDM317 | This study |
| GFP-Vps32 | *vps32* cDNA (DDB_G0275573) in pDM317 | This study |
| Vps4-GFP | *vps4* cDNA (DDB_G0284347) in pDM323 | This study |
| GFP-Atg8  RFP-Vps32 | *vps32* cDNA (DDB_G0275573) in pJSK421 (pDM410 GFP-Atg8a shuttle vector gateaway) | This study |
| pDNeoGFP-Plin | GFP-Plin | [6] |
| AmtA-mCherry | *amtA* cDNA(DDB_G0277503) in pDM1044 | [7] |
| Mycobacteria plasmids | | |
| pCherry10 | mCherry under control of the G13 promoter, Hyg^r^ | [8] |
| pMV306**::***lux* | bacterial luciferase under control of the G13 promoter, Kan^r^ | [9] |

**Supplementary references**

1. Cardenal-Munoz E, Arafah S, Lopez-Jimenez AT, Kicka S, Falaise A, Bach F, et al. Mycobacterium marinum antagonistically induces an autophagic response while repressing the autophagic flux in a TORC1- and ESX-1-dependent manner. PLoS Pathog. 2017;13(4):e1006344.

2. Mattei S, Ryves WJ, Blot B, Sadoul R, Harwood AJ, Satre M, et al. Dd-Alix, a conserved endosome-associated protein, controls Dictyostelium development. Dev Biol. 2005;279(1):99-113.

3. Aubry L, Mattei S, Blot B, Sadoul R, Satre M, Klein G. Biochemical characterization of two analogues of the apoptosis-linked gene 2 protein in Dictyostelium discoideum and interaction with a physiological partner in mammals, murine Alix. J Biol Chem. 2002;277(24):21947-54.

4. Veltman DM, Akar G, Bosgraaf L, Van Haastert PJ. A new set of small, extrachromosomal expression vectors for Dictyostelium discoideum. Plasmid. 2009;61(2):110-8.

5. King JS, Veltman DM, Insall RH. The induction of autophagy by mechanical stress. Autophagy. 2011;7(12):1490-9.

6. Du X, Barisch C, Paschke P, Herrfurth C, Bertinetti O, Pawolleck N, et al. Dictyostelium lipid droplets host novel proteins. Eukaryot Cell. 2013;12(11):1517-29.

7. Barisch C, Paschke P, Hagedorn M, Maniak M, Soldati T. Lipid droplet dynamics at early stages of Mycobacterium marinum infection in Dictyostelium. Cell Microbiol. 2015;17(9):1332-49.

8. Carroll P, Schreuder LJ, Muwanguzi-Karugaba J, Wiles S, Robertson BD, Ripoll J, et al. Sensitive detection of gene expression in mycobacteria under replicating and non-replicating conditions using optimized far-red reporters. PLoS One. 2010;5(3):e9823.

9. Andreu N, Zelmer A, Fletcher T, Elkington PT, Ward TH, Ripoll J, et al. Optimisation of bioluminescent reporters for use with mycobacteria. PLoS One. 2010;5(5):e10777.
