# Supplementary figures and images for: The ESCRT and autophagy machineries cooperate to repair ESX-1-dependent damage at the Mycobacterium-containing vacuole but have opposite impact on containing the infection
Source: PLoS Pathog. 2018 Dec 31;14(12):e1007501. doi: 10.1371/journal.ppat.1007501 (PMC6329560; doi:10.1371/journal.ppat.1007501)

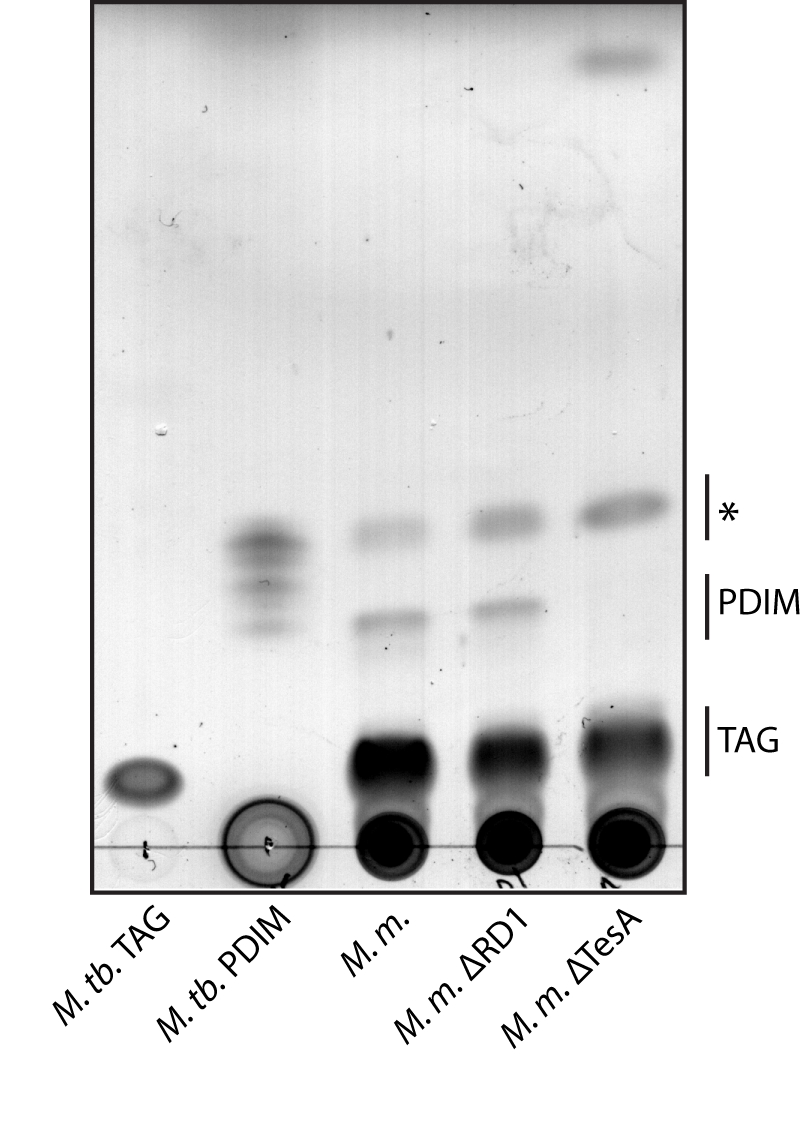

Supplement: S1 Fig — Lipids from M. marinum wt and M. marinum ΔRD1 were analysed by Thin Layer Chromatography (TLC). Presence of PDIMs was detected in both strains, in contrast to the PDIM defective mutant M. marinum ΔTesA used as a control [71]. The asterisk marks a lipid of unknown nature. (TIF) [file ppat.1007501.s002.tif]

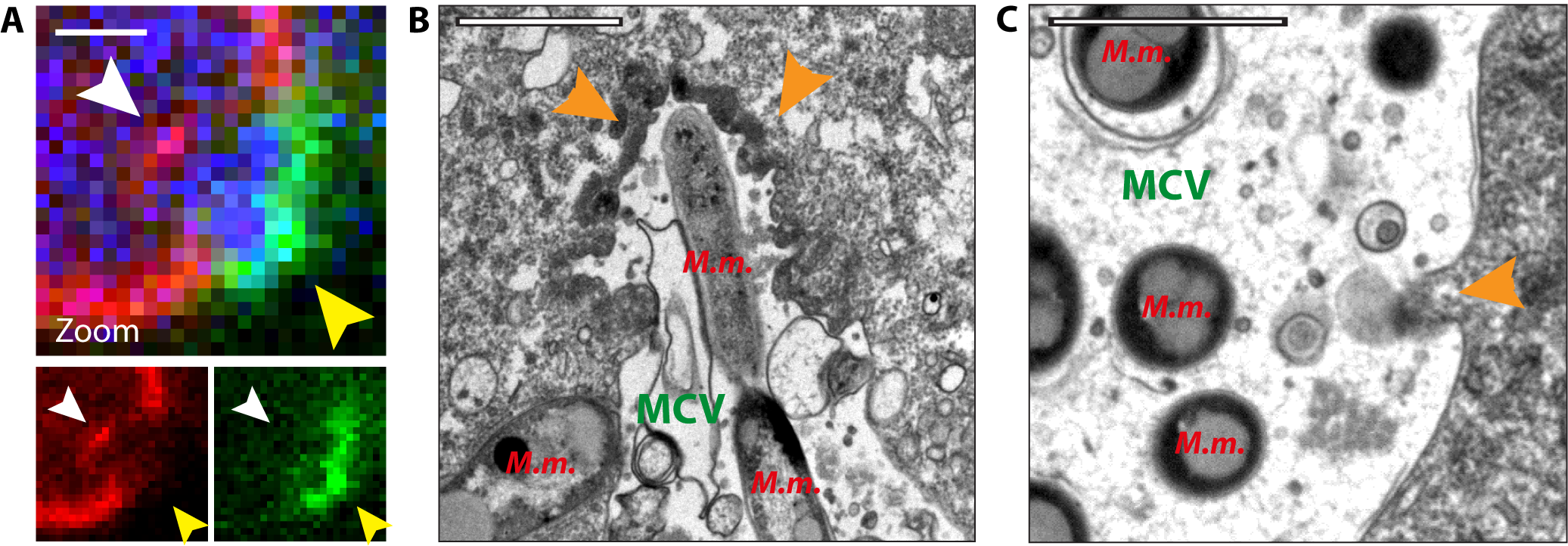

Supplement: S2 Fig — (A) High magnification inset of the picture shown in Fig 3B 31 hpi, to highlight the topology of GFP-Vps32 recruitment (yellow arrow) at the damaged MCV (labelled with AmtA-mCherry). The membrane of the compartment seems to invaginate towards the lumen of the MCV (white arrow). (B-I). D. discoideum cells were infected with M. marinum (“M.m.” labelled in red) and fixed for TEM to visualize the ultrastructure of the MCV. (B-C) Membrane invaginations at the MCV covered by electron-dense cytosolic material are highlighted with orange arrows. Image in (C) is a high magnification of Fig 1E in [23]. Scale bars, 1 μm. (TIF) [file ppat.1007501.s003.tif]

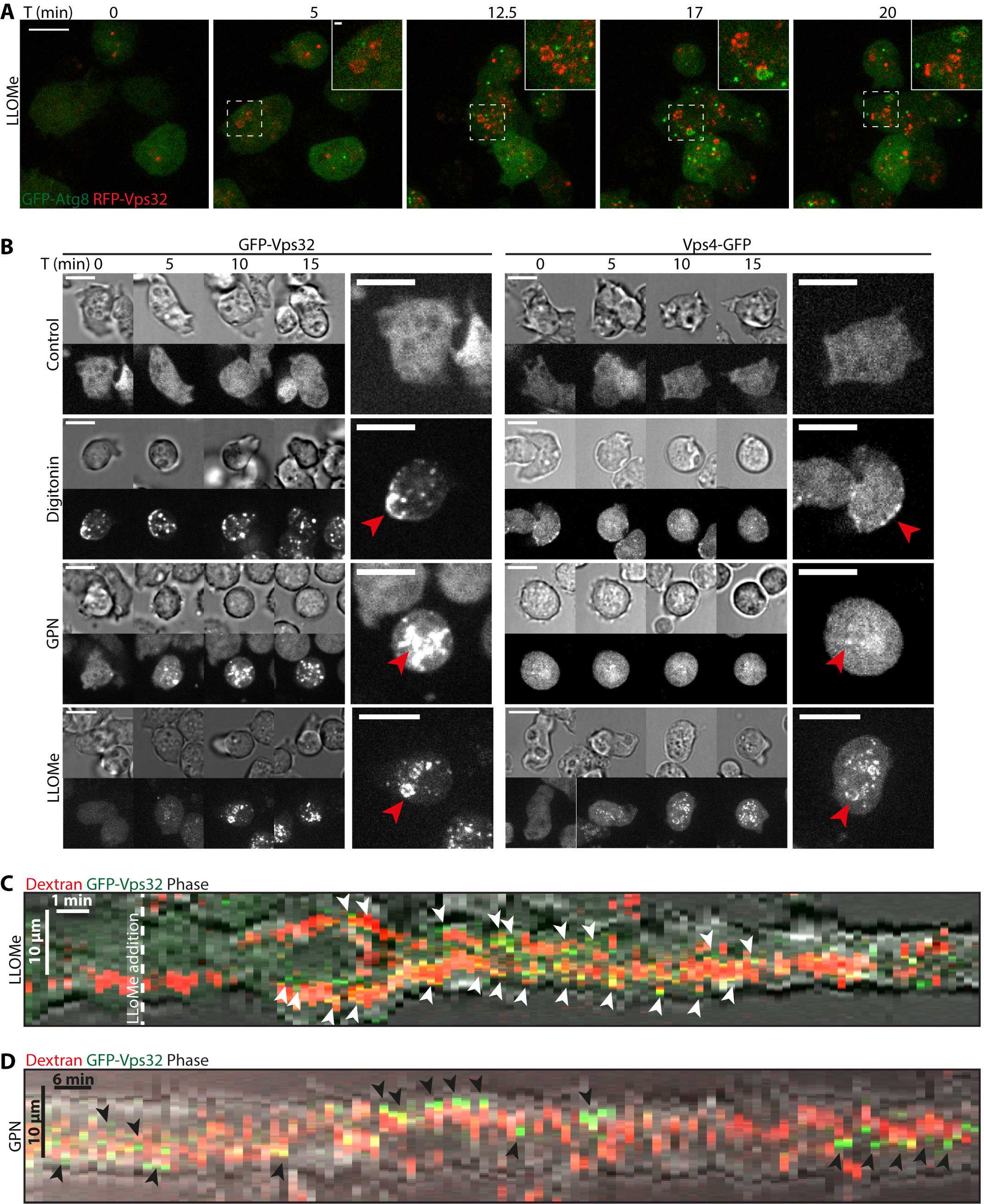

Supplement: S3 Fig — (A) D. discoideum cells co-expressing GFP-Atg8 and RFP-Vps32 were treated with LLOMe and monitored by time-lapse microscopy. Circular RFP-Vps32 structures were observed several minutes before GFP-Atg8 became apparent (see also S6 Movie). (B) D. discoideum expressing GFP-Vps32 or Vps4-GFP were treated with digitonin, GPN, LLOMe, or medium (control) and visualized over time. Still images show representative cells in phase-contrast and fluorescence at 0, 5, 10 and 15 min after treatment. On the right, magnification of one of the images per treatment. Red arrows point to GFP-Vps32 and Vps4-GFP structures at the sites of damage. Scale bars 10 μm. D. discoideum expressing GFP-Vps32 were incubated with TRITC-Dextran (red) (C) or Alexa Fluor 647 Dextran (red) (D) for at least 3 h to label all endosomes, treated with LLOMe or GPN, respectively, and monitored by time-lapse microscopy. Kymographs generated by a repeated linescan through a representative cell show the sustained association of GFP-Vps32 structures with the lysosomes and endosomes (black and white arrows). In D, the compound was added immediately before imaging started. (TIF) [file ppat.1007501.s004.tif]

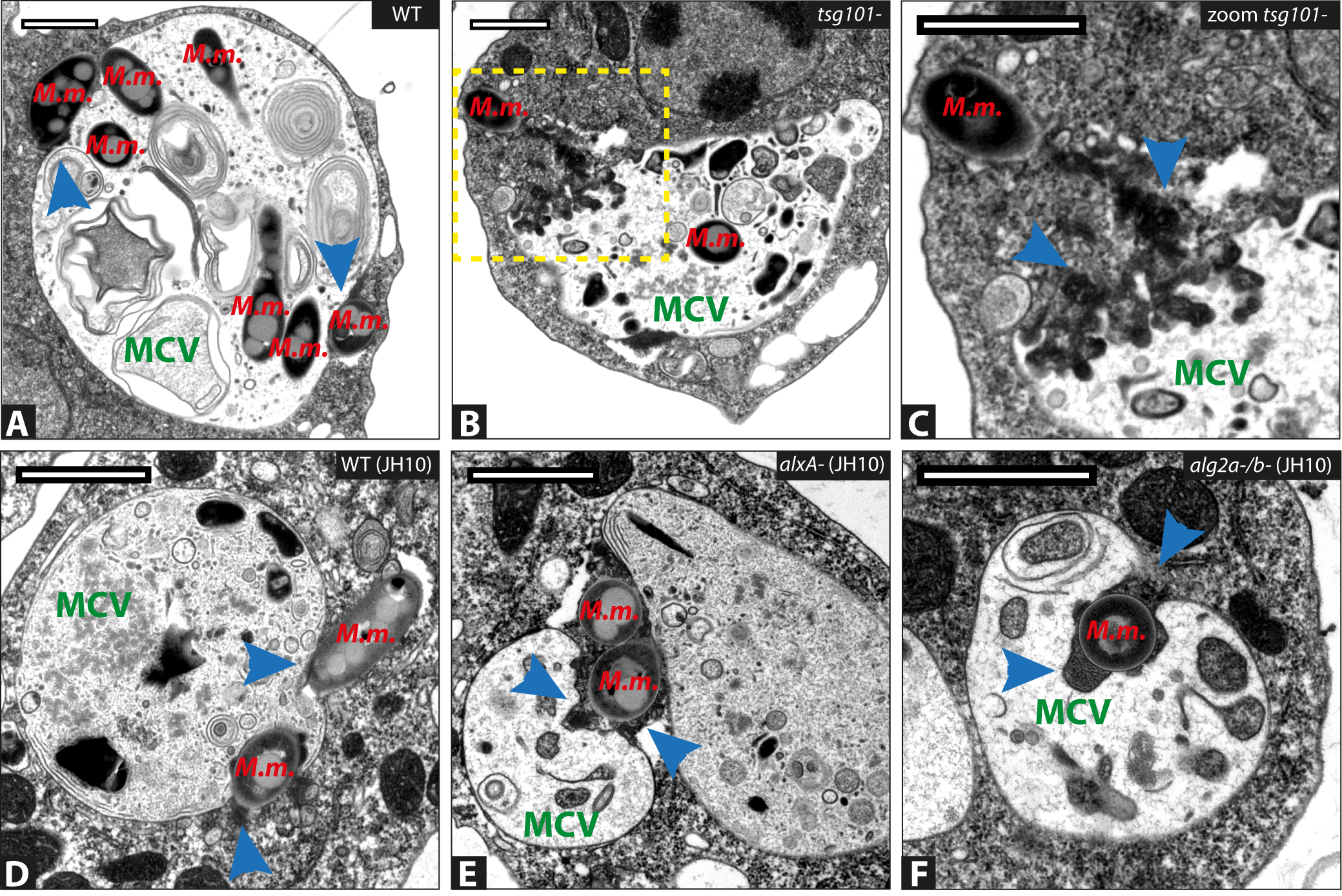

Supplement: S4 Fig — D. discoideum cells were infected with M. marinum and fixed for TEM at 24 hpi. M. marinum (“M.m.” labelled in red) accessed the cytosol in wt (A and D), tsg101- (B and C), alix- (E) and alg2a-/b- (F). Sites of membrane disruption are highlighted with blue arrows. (C) High magnification inset of the region of interest in (B). Scale bars, 1 μm. (TIF) [file ppat.1007501.s005.tif]

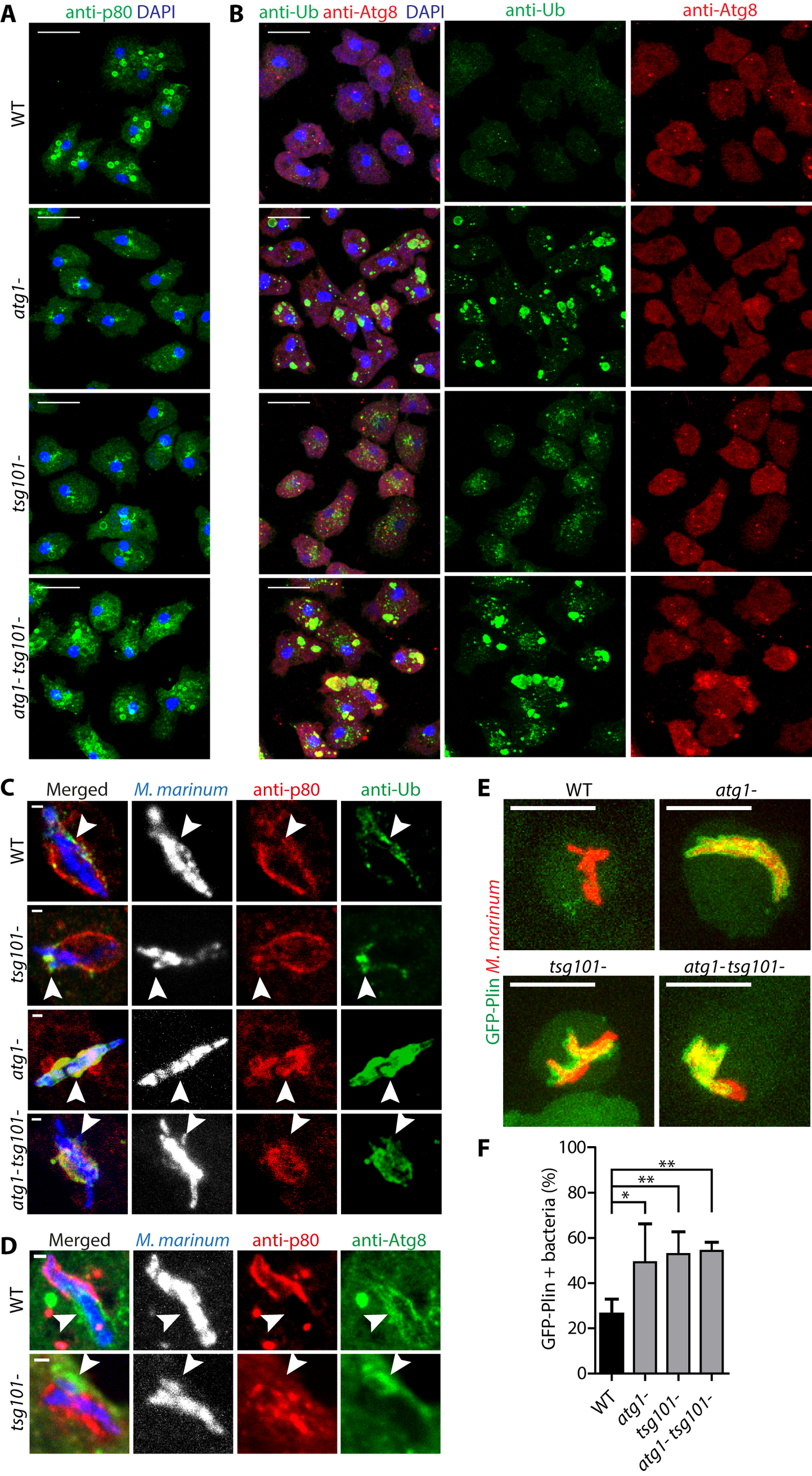

Supplement: S5 Fig — (A-B) D. discoideum wt or mutant (atg1-, tsg101- or atg1- tsg101-) were fixed for immunofluorescence. Post-lysosomes were labelled with p80 (green), nuclei were labelled with DAPI (blue), ubiquitin was in green and Atg8 in red. (C-D) D. discoideum wt or mutant (atg1-, tsg101-, or atg1-tsg101-) were infected with M. marinum (blue) and fixed for immunofluorescence. Both ubiquitinin and Atg8 (green) decorated the bacteria when the MCV (p80, red) was disrupted. Scale bars 1 μm. (E) D. discoideum wt or mutant (atg1-, tsg101-, or atg1-tsg101-) expressing GFP-Plin (green) were infected with M. marinum for live microscopy. All mutants showed an increase of GFP-Plin recruitment on the bacteria (red). (F) Proportion of the M. marinum bacteria or microcolonies decorated with GFP-Plin. The plot shows the mean and standard deviation (WT N = 4, n = 139; atg1- N = 3, n = 148; tsg101- N = 3, n = 98; atg1- tsg101- N = 3, n = 191). ANOVA and post hoc Fisher’s LSD test were performed. (TIF) [file ppat.1007501.s006.tif]

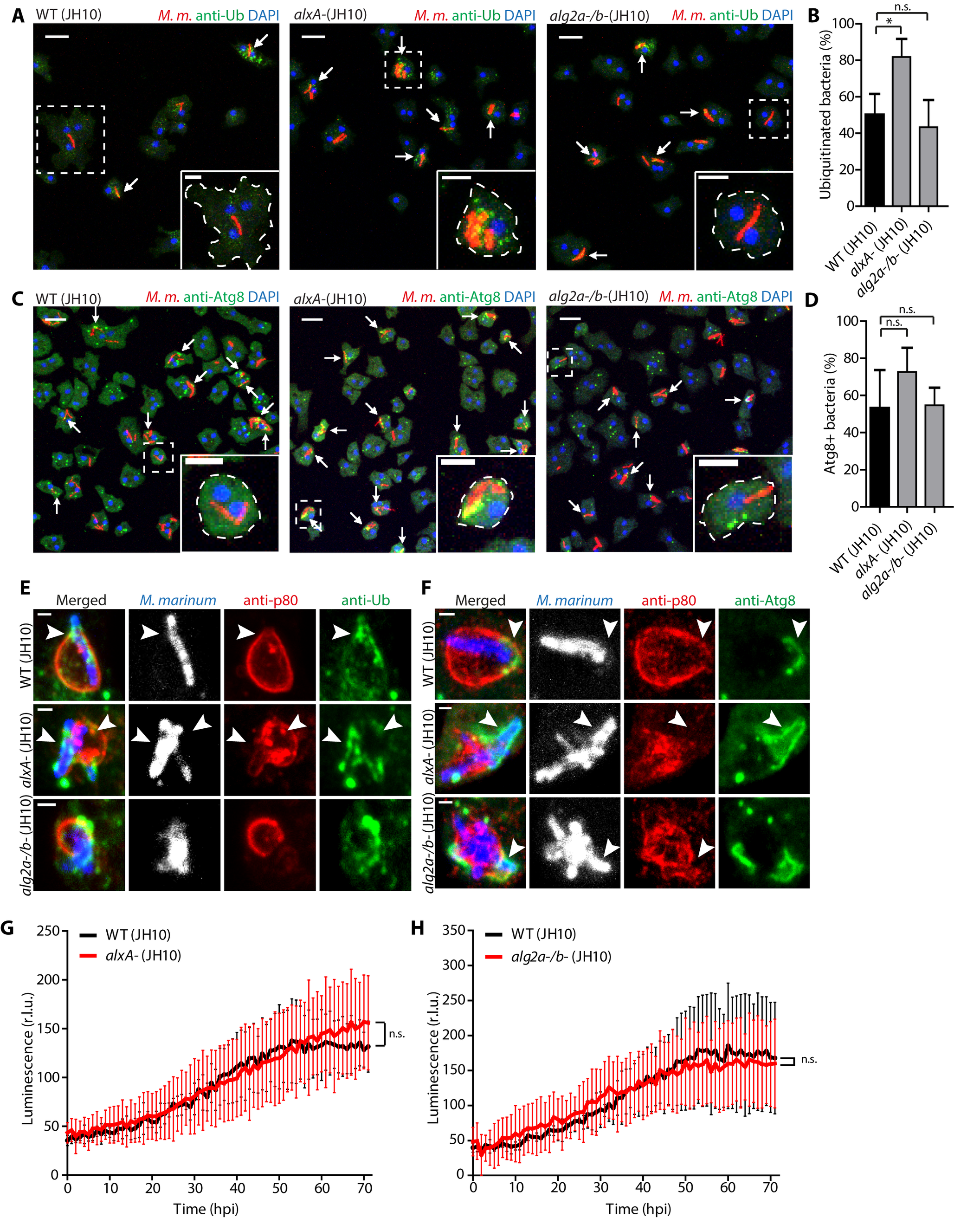

Supplement: S6 Fig — (A) D. discoideum wt or mutant (alxA- or alg2-/b-) were infected with M. marinum and fixed for immunostaining at 8 hpi (M. marinum in red, ubiquitin in green, DAPI in blue). Arrows point to ubiquitinated bacteria. Scale bars, 10 μm and 5 μm for the insets. (B) Quantification of the proportion of ubiquitinated bacteria or bacterial microcolonies. The plot shows the mean and standard deviation [WT (JH10) N = 4, n = 144; alxA- (JH10) N = 3, n = 134; alg2a-/b- (JH10) N = 3, n = 266]. (C) D. discoideum wt or mutant (alxA- or alg2-/b-) were infected with M. marinum and fixed for immunostaining at 8 hpi (M. marinum in red, Atg8 in green, DAPI in blue). Arrows point to bacteria decorated with Atg8. Scale bars, 10 μm and 5 μm for the insets. (D) Quantification of the proportion of bacteria or bacterial microcolonies decorated with Atg8. The plot shows the mean and standard deviation [WT (JH10) N = 4, n = 275; alxA- (JH10) N = 4, n = 170; alg2a-/b- (JH10) N = 4, n = 448]. ANOVA and post hoc Fisher’s LSD test were performed. (E-F) D. discoideum wt or mutant (alxA- or alg2-/b-) were infected with M. marinum (blue) and fixed for immunofluorescence. Both ubiquitinin and Atg8 (green) decorate the bacteria when the MCV (p80, red) was disrupted. Scale bars, 1 μm. (G-H) D. discoideum wt or mutant (alxA- or alg2-/b-) were infected with luminescent M. marinum and intracellular bacterial growth was monitored in a plate reader over 72 hpi. There was no significant difference between M. marinum growth in wt and mutants. Plots represent the mean and standard deviation of N = 3 independent experiments. Two-way ANOVA and post hoc Fisher’s LSD test were performed. (TIF) [file ppat.1007501.s007.tif]

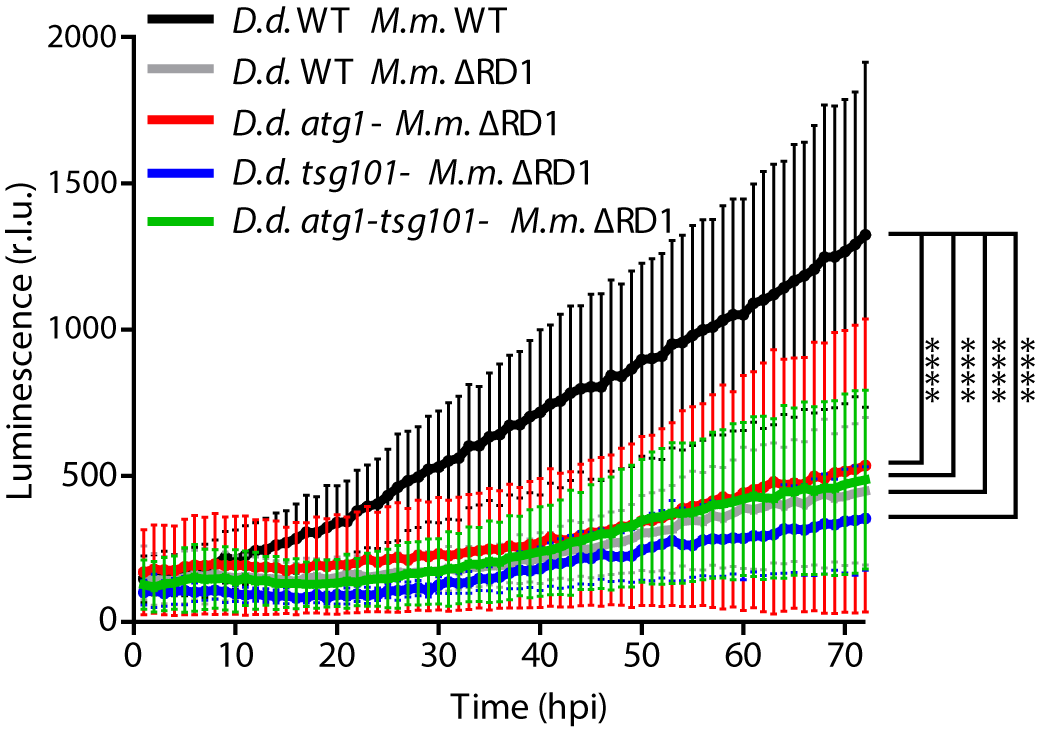

Supplement: S7 Fig — D. discoideum wt or mutant (atg1-, tsg101- or atg1-tsg101-) were infected with luminescent M. marinum (wt or ΔRD1) and intracellular bacterial growth was monitored in a plate reader over 72 hpi. In all D. discoideum mutants tested, M. marinum ΔRD1 growth was significantly attenuated compared to M. marinum wt in a D. discoideum wt host. Plots represent the mean and standard deviation of N = 3 independent experiments. Two-way ANOVA and post hoc Fisher’s LSD test were performed. (TIF) [file ppat.1007501.s008.tif]
